# *De novo* assembly of a Tibetan genome and identification of novel structural variants associated with high-altitude adaptation

**DOI:** 10.1093/nsr/nwz160

**Published:** 2019-10-23

**Authors:** Ouzhuluobu, Yaoxi He, Haiyi Lou, Chaoying Cui, Lian Deng, Yang Gao, Wangshan Zheng, Yongbo Guo, Xiaoji Wang, Zhilin Ning, Jun Li, Bin Li, Caijuan Bai, Baimakangzhuo, Gonggalanzi, Dejiquzong, Bianba, Duojizhuoma, Shiming Liu, Tianyi Wu, Shuhua Xu, Xuebin Qi, Bing Su

**Affiliations:** 1 High Altitude Medical Research Center, School of Medicine, Tibetan University, Lhasa 850000, China; 2 State Key Laboratory of Genetic Resources and Evolution, Kunming Institute of Zoology, Chinese Academy of Sciences, Kunming 650223, China; 3 Chinese Academy of Sciences Key Laboratory of Computational Biology, CAS-MPG Partner Institute for Computational Biology, Shanghai Institute of Nutrition and Health, Shanghai Institutes for Biological Sciences, University of Chinese Academy of Sciences, Shanghai 200031, China; 4 Center for Excellence in Animal Evolution and Genetics, Chinese Academy of Sciences, Kunming 650223, China; 5 Fukang Obstetrics, Gynecology and Children Branch Hospital, Tibetan Fukang Hospital, Lhasa 850000, China; 6 Center for Disease Control, Tibet Autonomous Region, Lhasa 850000, China; 7 National Key Laboratory of High Altitude Medicine, High Altitude Medical Research Institute, Xining 810012, China; 8 School of Life Science and Technology, Shanghai Tech University, Shanghai 201210, China; 9 Collaborative Innovation Center of Genetics and Development, Shanghai 200438, China; 10 Kunming College of Life Science, University of Chinese Academy of Sciences, Beijing 100101, China

**Keywords:** long-read sequencing, Tibetan, structural variants, genetic adaptation, reference genome

## Abstract

Structural variants (SVs) may play important roles in human adaptation to extreme environments such as high altitude but have been under-investigated. Here, combining long-read sequencing with multiple scaffolding techniques, we assembled a high-quality Tibetan genome (ZF1), with a contig N50 length of 24.57 mega-base pairs (Mb) and a scaffold N50 length of 58.80 Mb. The ZF1 assembly filled 80 remaining N-gaps (0.25 Mb in total length) in the reference human genome (GRCh38). Markedly, we detected 17 900 SVs, among which the ZF1-specific SVs are enriched in GTPase activity that is required for activation of the hypoxic pathway. Further population analysis uncovered a 163-bp intronic deletion in the *MKL1* gene showing large divergence between highland Tibetans and lowland Han Chinese. This deletion is significantly associated with lower systolic pulmonary arterial pressure, one of the key adaptive physiological traits in Tibetans. Moreover, with the use of the high-quality *de novo* assembly, we observed a much higher rate of genome-wide archaic hominid (Altai Neanderthal and Denisovan) shared non-reference sequences in ZF1 (1.32%–1.53%) compared to other East Asian genomes (0.70%–0.98%), reflecting a unique genomic composition of Tibetans. One such archaic hominid shared sequence—a 662-bp intronic insertion in the *SCUBE2* gene—is enriched and associated with better lung function (the FEV1/FVC ratio) in Tibetans. Collectively, we generated the first high-resolution Tibetan reference genome, and the identified SVs may serve as valuable resources for future evolutionary and medical studies.

## Introduction

Next-generation sequencing (NGS) is a powerful tool to study human genomic variations through simple alignment of short reads to a reference genome. However, short reads have unavoidable limitations for genome assembly, especially for detection of structural variants (SVs) that have been shown to play an important role in normal and abnormal human biology [1,2]. By contrast, with an advantage of long reads (>10 kilo-base pair, kb), the single-molecular real-time (SMRT) sequencing (also called the third-generation sequencing, TGS) has been proven effective in resolving complex genomic regions, such as sequences with SVs [3,4]. Meanwhile, the application of next-generation mapping technologies provides complementary approaches to *de novo* genome assembly, including BioNano, 10X Genomics and Hi-C, etc. Recently, with the aid of SMRT sequencing and next-generation mapping methods, two long-read Asian genome assembles (AK1 and HX1) were released [5,6].

Tibetans represent a unique highland population permanently living at the Tibetan Plateau (average elevation: >4000 m)—one of the most extreme environments on Earth. Their permanent settlement in the Qinghai-Tibetan plateau was dated as early as 30 000 years ago based on genetic data [7–9]. Previous genetic studies have identified two key genes (*EPAS1* and *EGLN1*) carrying adaptive alleles that help maintain relatively lower hemoglobin concentration in native Tibetans so that over-production of red cells (polycythemia) at high altitude could be avoided [10–20]. Also, a Tibetan-enriched 3.4-kb deletion (TED) near *EPAS1* was reported [21]. Additionally, it was proposed that the Tibetan-enriched *EPAS1* variants were inherited from Denisovan-like hominid [22]. This evidence suggests that the high-altitude adaptation of Tibetans is probably multi-faceted, involving different types of genomic variations.

Besides hemoglobin concentration, there are other key adaptive physiological traits in Tibetans, such as elevated resting ventilation, low hypoxic pulmonary vasoconstrictor response [23] and lower blood nitric-oxide levels [24], which cannot be fully explained by the known single nucleotide variations (SNVs) identified using NGS data. Putatively, SVs located in the regulatory regions of the genome may contribute to these unresolved adaptive traits. Also, the sequences present in Tibetans but absent in the human reference genome are putative introgressions from archaic humans, which have not been systematically evaluated. Hence, these unsolved questions call for a high-quality Tibetan reference genome.

We combined SMRT long-read sequencing with multiple scaffolding techniques, as well as short-read deep-sequencing, and we *de novo* assembled a high-quality Tibetan genome (ZF1). The assembled Tibetan genome reached a contig N50 size of 24.57 Mb and a scaffold N50 size of 58.80 Mb. We used a read-mapping approach to detect SVs in the assembled ZF1 genome. By comparing with two previous long-read Asian genome assemblies (AK1 and HX1), we identified a large number of novel SVs, some of which are enriched in Tibetans and showed association with pulmonary arterial pressure and lung functions. Furthermore, using the high-quality ZF1 assembly, we found a much higher rate of genome-wide archaic hominid (Altai Neanderthal and Denisovan) shared non-reference sequences in ZF1 than in other East Asian genomes.

**Figure 1.**
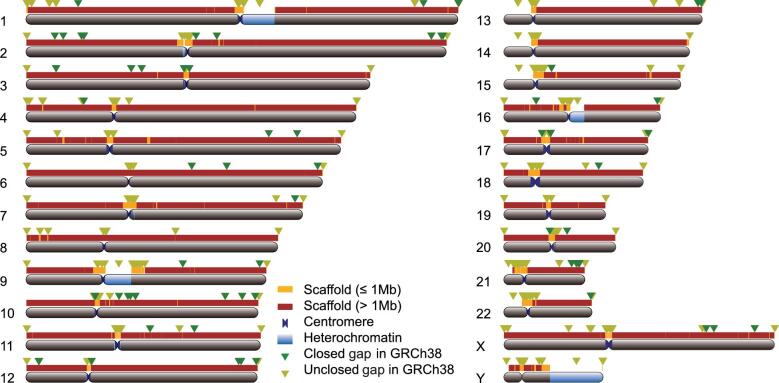
*De novo* assembly of the ZF1 genome compared to GRCh38. Scaffold coverage and gap closure over GRCh38 per chromosome are shown in the plot. The colored bars above each chromosome represent the ZF1 scaffolds, with the dark red segments for the long scaffolds (> 1 Mb) and the orange segments for the short ones (≤ 1 Mb). Closed euchromatic gaps are labeled by the green triangles on each chromosome, and the unclosed gaps in ZF1 by the khaki-green triangles.

## Results

### *De novo* assembly of the Tibetan genome and gap filling on the reference genome

We performed SMRT long-read sequencing using PacBio RSII at 70× coverage and obtained a total of 24.9 M subreads with median and mean read length of 9.5 kb and 10.3 kb, respectively (Supplementary Fig. 1). The long reads were error-corrected and assembled into contigs by Falcon, and then the assembled contigs were polished by Quiver [25]. In total, we generated 3148 contigs with a N50 length of 23.62 Mb (Supplementary Fig. 2 and Supplementary Tables 1–3, Methods). To order and link these contigs into larger scaffolds, we utilized the data of BioNano and 10X Genomics, and constructed two versions of scaffolding (Supplementary Tables 2 and 3). The first version used the 10X Genomics linked-reads (100×) to link the contigs into larger scaffolds and then combined the physical maps with unique motifs from BioNano. This scaffolding strategy resulted in 2403 scaffolds with a N50 length of 45.42 Mb (Supplementary Fig. 2a and Supplementary Table 2, Methods). The second version used the BioNano data first and then the 10X Genomics reads, which resulted in 2321 scaffolds with a N50 length of 47.17 Mb (Supplementary Fig. 2b and Supplementary Table 3). Considering the longer scaffold N50 length, we chose the second version for further improvement using Hi-C data.

**Table 1.** Comparison of the ZF1 *de novo* assembly with the published human genome assemblies.

Assembly	Assembly approach	Sequencing platform	Contig N50 (Mb)	Scaffold N50 (Mb)	Genome size (Gb)	Gaps number	Gap length (Mb)
ZF1	WGS, Hi-C	PacBio, BioNano, 10X Genomics, Hi-C, Illumina-PE	24.57	58.8	2.9	740	7.82
HX1	WGS	PacBio, BioNano, Illumina-PE	8.33	21.98	2.93	10 901	39.34
AK1	WGS, BAC	PacBio, BioNano, Illumina-PE	17.92	44.85	2.9	264	37.34
GRCh38	BAC, Fosmid	Sanger, FISH, OM, fingerprint contigs	56.41	67.79	3.2	940	159.97
NA12878(ASM101398)	WGS	PacBio, BioNano	1.56	26.83	3.2	2332	146.35

WGS, whole-genome sequencing; BAC, bacterial artificial chromosome; PE, pair end; FISH, fluorescence *in situ* hybridization; OM, optical mapping.

We generated 100× Hi-C data of ZF1 (Supplementary Table 1). Using SALSA [26], we grouped the scaffolds using Hi-C data, leading to a final version of the assembled ZF1 genome of 2.89 Gb with a contig N50 length of 24.57 Mb and a scaffold N50 length of 58.80 Mb. In addition, we performed short-read sequencing using an Illumina HiSeq X10 platform and generated 100× coverage of the ZF1 genome to improve base-level accuracy (Fig. 1 and Supplementary Tables 2 and 3, Methods). We also generated a phased version of the ZF1 genome assembly (see Methods for technical details).

Next, we used the *de novo* assembled ZF1 genome to conduct gap closure for the human reference genome GRCh38. A total of 80 of the 940 N-gaps in the GRCh38 human reference genome were completely filled by the ZF1 assembly and the total length was 0.25 Mb (Fig. 1 and Supplementary Table 4, Methods).

To evaluate the completeness and accuracy of the ZF1 genome assembly, we compared the ZF1 assembly with two previous long-read Asian genome assemblies (AK1 [5] and HX1 [6]) and a high-quality European genome (ASM101398, sample ID: NA12878 [27]). We found that the total bases (non-N bases in assembly) of the three Asian *de novo* assemblies (ZF1, AK1 and HX1) were quite similar (Table 1 and Supplementary Fig. 3). Notably, the remaining gap length of ZF1 (7.82 Mb) is much shorter than those in AK1 (37.34 Mb) and HX1 (39.34 Mb) (Supplementary Table 5). In addition, using MUMmer [28], we assessed the consensus quality of the ZF1 assembly by aligning the ZF1 chromosomes with those of GRCh38 and we obtained 99.90% consensus accuracy for the ZF1 assembly, which is better than those for HX1 (99.73%), YH2.0 (99.81%), NA12878 (99.73%) and HuRef (99.84%) [6] (Supplementary Fig. 4).

Additionally, we evaluated the base-error rate of the ZF1 assembly using our 100× Illumina short-read data with the previous approach [29,30]. The inconsistency rate is 0.0006% (Supplementary Table 6)—well below one error per 10 000 bases, the quality standard used for human genome [31].

Furthermore, we annotated the ZF1 assembly using CESAR2.0 [32] and made a functional annotation for ZF1 genes with four databases (KEGG, Swiss-Prot, InterPro and NR) (Supplementary Fig. 5). We obtained a similar number of annotated genes compared with GRCh38 (ZF1: 19 805 vs GRCh38: 19 267) and 99.8% of the ZF1 genes were annotated by multiple databases. Notably, the ZF1 assembly embraced a longer average length of coding sequence (CDS) than GRCh38 (Supplementary Fig. 6 and Supplementary Tables 7 and 8) and this improvement may stem from long-read assembly of more exons of the ZF1 assembly. Taken together, our ZF1 assembly provided a reliable reference genome for downstream analyses.

### Profiling SVs of the ZF1 genome and evolutionary genetics analysis

**Figure 2.**
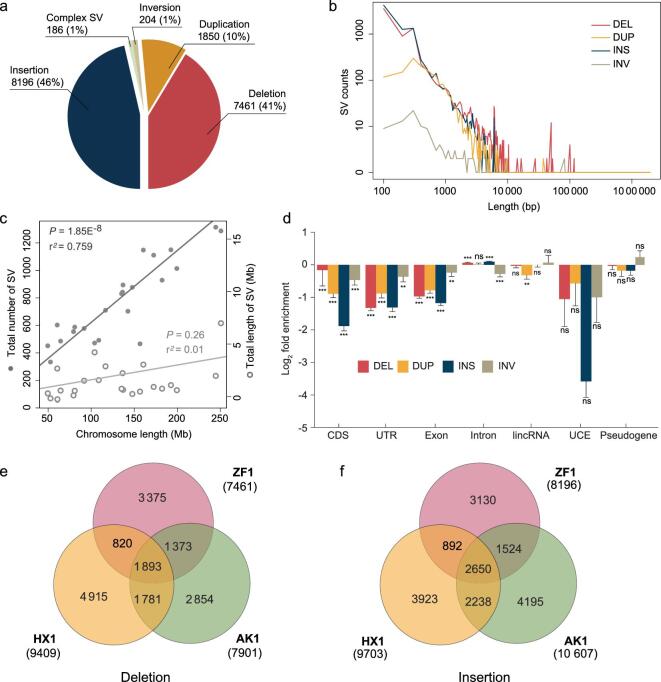
A summary of structural variants (SVs) detected in ZF1. (a) Pie plot shows the number and the proportion of insertions, deletions, duplications, inversions and complex SVs detected in ZF1. (b) Size distribution of SVs in ZF1. (c) Correlation of chromosome length with SV numbers (dark gray) and SV lengths (light gray). (d) Enrichment/depletion of genomic elements for different SV classes. CDS, coding sequences; UTR, untranslated region; UCE, ultra-conserved element. *P*-value refers to the significance of permutation test. ***P < 0.001; **P < 0.01; ns, non-significant (*P* > 0.05). (e) and (f) Overlap of SVs among ZF1, HX1 and AK1 for deletions (e) and insertions (f), respectively. An overlapped SV was defined as one with overlapping length reaching at least 50% of reciprocal similarity.

We employed a read-mapping-based approach to call SVs from the ZF1 PacBio long reads (see Methods for details) [4]. For the compatibility of conducting downstream SV comparison analysis with the public data, we used the human genome build GRCh37 instead of GRCh38 as the reference. Within the size range of 50 bp to 2 Mb, we obtained 17 900 SVs, including 7461 deletions, 1853 duplications, 8196 insertions, 204 inversions and 186 complex SVs. We found 75% of the SVs were supported by results from other platforms (i.e. Illumina X10, BioNano and 10X Genomics; Fig. 2a and Supplementary Tables 9 and 10). The majority (89%) of the SVs are smaller than 1 kb, with the median lengths for deletion, insertion, duplication and inversion being 166, 144, 543 and 1399 bp, respectively (Fig. 2b and Supplementary Fig. 7). The distribution of the large SVs (>1 kb) on the 24 chromosomes (including X and Y chromosomes) of ZF1 is shown in Supplementary Fig. 8. The SVs cover 57.8 Mb in total, accounting for ∼2% of the entire genome (Fig. 2b). Almost 70% of the SVs contain repetitive elements such as SINEs, LINEs, simple repeats and satellites, etc. (Supplementary Table 11). Besides, we found the number of SVs in each chromosome was significantly correlated with chromosome length (*R*^2^ = 0.759, *P* = 1.85E^−8^) while the SV length was not (*R*^2^ = 0.01, *P* = 0.26) (Fig. 2c and Supplementary Fig. 9).

To explore the genomic features of these SVs, we analysed the intersection of SVs with various functional genomic elements, including CDSs, untranslated regions (UTRs), exons, introns, lincRNAs, ultra-conserved elements (UCEs) and pseudogenes. We found a significant depletion (*P* < 0.001; permutation test) of genomic elements for different SV classes compared to a random background, suggesting a selective constraint of SVs as previously indicated [2]. Additionally, combining the SVs of ZF1, AK1 and HX1, we used Watterson’s θ and estimated the mutation rates for deletion and insertion to be 0.186 and 0.203 per generation per haploid genome, respectively (Supplementary Table 12). These estimations are higher than the previous report based on NGS data [2], likely due to the increased power of the TGS platform in detecting SVs.

Among the 17 900 SVs of ZF1, 6505 (36.3%) were not found in either AK1 or HX1, including 3375 deletions and 3130 insertions, accounting for 45.24% (3375/7461) and 38.19% (3130/8196) of the total deletions and insertions, respectively (Fig. 2e and f). Genes located <5 kb downstream or upstream of these ZF1-specific SVs (annotated using Variant Effect Predictor (VEP)) were defined as ZF1-specific-SV-associated genes (ZSAGs). Totally, we found 1832 ZSAGs enriched in four functional clusters, i.e. positive regulation of GTPase activity (false discovery rate, FDR = 1.78E^−5^), intracellular signal transduction (FDR = 0.008), transmembrane receptor protein tyrosine kinase signaling pathway (FDR = 0.011) and peptidyl-tyrosine phosphorylation (FDR = 0.035) (Supplementary Fig. 10 and Supplementary Table 13).

We next explored how many ZSAGs were related to hypoxic regulation. Among the 571 priori candidate genes of hypoxia adaptation in the Tibetans (473 known hypoxia-related genes [19] and 168 reported genes showing signals of Darwinian positive selection in Tibetans [10–15,17,33]), we found 69 of them overlapped with the 1832 ZSAGs (Supplementary Tables 14 and 15; odds ratio = 2.42, *P* = 3.40E^−12^, Chi-squared test). Interestingly, these newly identified hypoxia- and selection-related SVs are all located in either intronic or intergenic regions and, if functional, they are more likely to affect gene-expression regulation.

### Novel Tibetan-specific SVs are associated with high-altitude adaptation

The high-resolution ZF1 genome assembly provides high-confidence SVs with precise breakpoint positions, which could serve as a reference panel to assess the variant frequency difference between Tibetans and lowland populations. To this end, we analysed the genetic divergence of SVs between Tibetans and Han Chinese using the published NGS data of 38 Tibetans and 39 Han Chinese [8] (see Methods for details). We listed the 124 SVs (the top 5% of the 1887 copy number variants (CNVs) and 593 insertions that passed the NGS-genotyping filtering; see Methods) with the highest between-population divergence (measured by V_ST_) in Supplementary Tables 16 and 17. The most diverged SV was the previously reported TED near *EPAS1* (V_ST_ = 0.725) [21] (Supplementary Fig. 11 and Supplementary Table 16). The remaining 123 SVs contain 93 CNVs (Supplementary Fig. 11 and Supplementary Table 16) and 30 insertions (Supplementary Fig. 12 and Supplementary Table 17).

Notably, we found a 163-bp deletion (chr22: 40935468-40935631, hg19) with an allelic divergence of 0.227 between Tibetans and Han Chinese (the allele frequencies are 0.544 and 0.317 in Tibetans and Han Chinese, respectively) (Supplementary Table 18). The V_ST_ of this variant (0.106) is among the top 5% of the genome-wide SVs (5% cut-off = 0.0956), a suggestive signal of selection and less likely caused by genetic drift or other demographic events according to the simulation analysis (see Methods, Supplementary Fig. 13).

**Figure 3.**
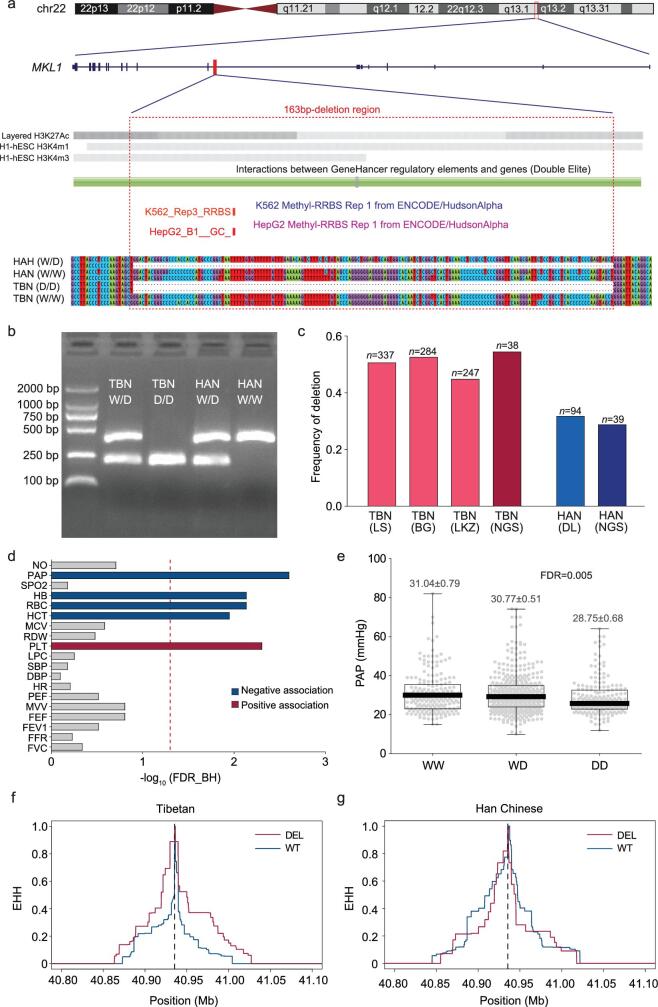
The Tibetan-enriched *MKL1* 163-bp deletion and its association with physiological traits. (a) The schematic map indicating the genomic location (upper panel), epigenetic signals (histone modification and DNA methylation, middle panel) and sequence alignment (bottom panel) of the *MKL1* deletion and its flanking sequences in Tibetans and Han Chinese. (b) Genotyping electromorphic of the *MKL1* deletion. The two alleles are indicated as ‘W’ (wild type) and ‘D’ (deletion). (c) Allele frequencies of the *MKL1* deletion in Tibetans (TBN) and Han Chinese (HAN); TBN (LS), Tibetans at Lhasa; TBN (BG), Tibetans at Bange; TBN (LKZ), Tibetans at Langkazi; HAN (DL), Han Chinese at Dalian; TBN (NGS) and HAN (NGS), Tibetans and Han Chinese from the NGS data (Methods). (d) Genetic association between the *MKL1* deletion and multiple physiological traits in Tibetans (*n* = 868). The dot line in red refers to the cut-off of statistical significance with false discovery rate (FDR) of ∼5% by Benjamini and Hochberg [42] and the trait abbreviations are described in Methods. (e) Comparison of pulmonary arterial pressure levels among three different genotypes at the *MKL1* deletion; W-wide type (non-deletion). *P*-value was calculated assuming an additive model with multiple-testing correction using Bejamini and Hochberg FDR control (FDR_BH) (Methods). (f) and (g) Estimation of EHH decay of haplotypes in Tibetans (f) and Han Chinese (g) surrounding the *MKL1* 163-bp deletion. The physical position of the 163-bp deletion is indicated by the vertical dashed line in (f) and (g).

This deletion is located in the intronic region of *MKL1*, which encodes Megakaryoblastic Leukemia 1 and was previously reported to regulate hypoxia-induced pulmonary hypertension in rodents [34,35]. We found that this 163-bp deletion overlapped with multiple histone modification signals (reported by ENCODE), suggesting that it is located in a region with enhancer activity. We also detected a GeneHancer regulatory element (MKL1/GH22J040443) and two methylation hotspots in this region (Fig. 3a and Supplementary Fig. 14).

We measured 19 physiological traits (varied blood, heart and lung indexes) and collected blood samples from 1039 indigenous adult Tibetans. Using PCR (polymerase chain reaction) and Sanger sequencing, we genotyped the *MKL1* deletion in 868 Tibetans from three geographic populations, including 337 unrelated individuals from Lhasa (elevation: 3658 m), 284 unrelated individuals from Bange (elevation: 4700 m) and 247 unrelated individuals from Langkazi (elevation: 5108 m). We also genotyped 94 unrelated Han Chinese from northern China (elevation: 60 m). The frequencies of the *MKL1* deletion are similar with those estimated based on NGS data (0.500 in Tibetans and 0.287 in Han Chinese; Fig. 3c and Supplementary Table 18, Methods). We next performed association analysis using the pooled Tibetan samples (*n* = 868), since no genetic heterogeneity was detected among the three Tibetan populations. We found that the *MKL1* deletion was negatively associated with PAP (systolic pulmonary arterial pressure) (FDR = 0.005, Fig. 3e) and the *MKL1* deletion carriers tend to have a lower PAP, consistently with the well-known low hypoxic pulmonary vasoconstrictor response in Tibetans [23]. Interestingly, the *MKL1* deletion also showed association with several blood indices, including negative associations with HB (hemoglobin concentration) (FDR = 0.03), HCT (hematocrit) (FDR = 0.02) and RBC (red blood count) (FDR = 0.01), and a positive association with PLT (platelets) (FDR = 0.005) (Fig. 3d and Supplementary Table 19), implying that the *MKL1* deletion might be involved in multiple regulatory effects on pulmonary and blood indexes. When using the most rigorous adjustment (19 traits × 4 SVs) for multiple test correction, PAP, RBC and PLT still remain significant: FDR (PAP) = 0.01, FDR (RBC) = 0.04 and FDR (PLT) = 0.01.

In addition to the *MKL1* deletion, we also selected other SV overlapping genes with previous evidence of positive selection or related with hypoxia regulation (Supplementary Table 20). Although these SVs were not among the top 5% diverged SVs between Tibetan and Han Chinese, some of them were significantly associated with multiple physiological traits (Supplementary Table 19). For example, a 53-bp insertion (allele frequency: 0.424 in TBN and 0.339 in HAN, Supplementary Table 20) in *COL6A2* was significantly associated with systolic blood pressure (SBP; *P* = 0.002, FDR = 0.03) and diastolic blood pressure (DBP; *P* = 0.005, FDR = 0.035) (Supplementary Figs 15 and 16 and Supplementary Table 19). *COL6A2* encodes eukaryotic translation initiation factor 4E with selection signals in Ethiopian high-altitude populations [36]. Another example is a 63-bp insertion in *EIF4E2*. The protein encoded by *EIF4F2* can form a complex with HIF-2 (encoded by *EPAS1*) and RBM4 under hypoxia as an oxygen-regulated switch [37]. This insertion (allele frequency: 0.457 in TBN and 0.382 in HAN) was significantly associated with maximum ventilatory volume (MVV) (*P* = 0.04, FDR = 0.293, Supplementary Fig. 15 and Supplementary Table 19).

**Figure 4.**
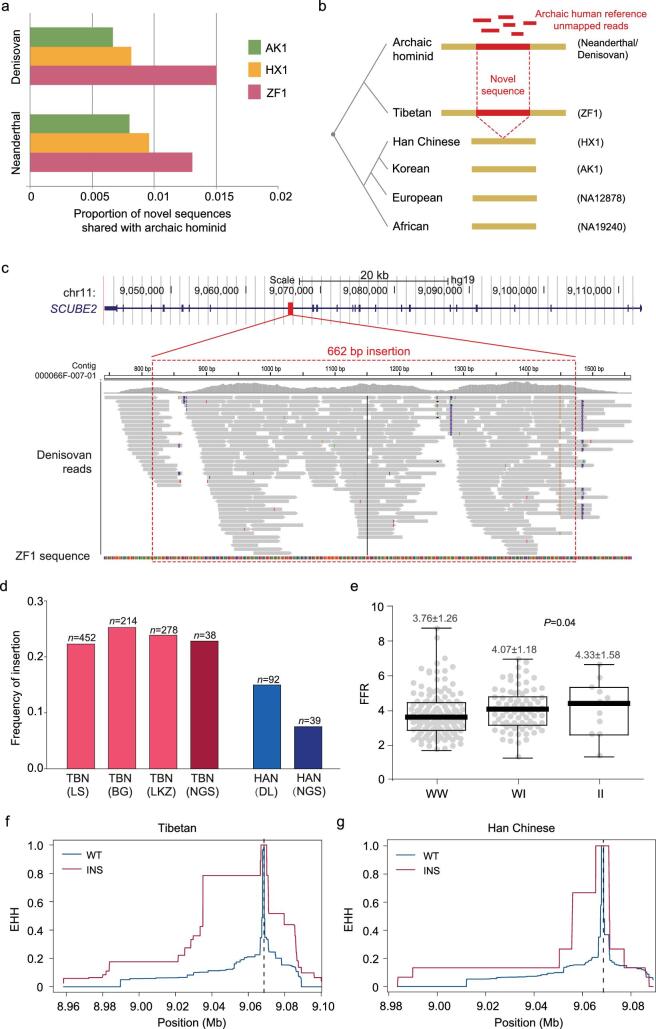
ZF1-speicific novel sequences shared with archaic hominins. (a) Genomic proportion of the non-reference sequences shared with archaic hominids in the three *de novo* assembled Asian genomes. (b) Schematic diagram of the ZF1-sepcific non-reference sequences shared with archaic hominids. Novel non-reference sequences (red) shared between Tibetan (ZF1) and archaic hominids but absent in the other representative modern human genomes (one European, one African and two Asian genomes). (c) A novel sequence shared between ZF1 and archaic hominid in the intron of *SCUBE2*. This sequence was found in both Denisovan and Altai Neanderthal. Shown here is the Denisovan reads mapping. The upper panel shows the position of this 662-bp novel sequence in the human reference genome GRCh37. The bottom panels indicate the Denisovan reference unmapped reads aligned to the ZF1 contig. (d) Allele frequencies of the 662-bp insertion in Tibetans (TBN) and Han Chinese (HAN). TBN (LS), Tibetans at Lhasa; TBN (BG), Tibetans at Bange; TBN (LKZ), Tibetans at Langkazi; HAN (DL), Han Chinese at Dalian; TBN (NGS) and HAN (NGS), insertion frequency estimated by next-generation-sequencing data (Methods). (e) Comparison of the FEV1/FVC ratios among three different genotypes at the *SCUBE2* insertion; I, insertion; W, wide type (non-insertion). (f) and (g) Estimation of EHH in TBN (f) and HAN (g) around the *SCUBE2* insertion at Chr11:9068607. The physical position of the insertion is indicated by the vertical dashed line in (f) and (g).

### Identification of ZF1-specific novel sequences shared with archaic humans

Previous studies have found evidence of Denisovan-like archaic introgression in the Tibetan genome such as the 32.7-kb fragment in *EPAS1* [22] and a ∼300-kb region in the chromosome 2 derived from unresolved archaic ancestry [8]. However, the sequences that are not included in the human reference genome but present in both archaic and Tibetans have not been systematically assessed. Taking advantage of the *de novo* ZF1 assembly, we performed a genome-wide search of archaic-sharing non-reference sequences (NRSs) and compared the results with the two *de novo* assembled Asian genomes (AK1 and HX1) (Methods). We found a total length of 39.6-Mb ZF1 sequences shared with the Altai Neanderthal genome and 45.9-Mb ZF1 sequences shared with the Denisovan genome, corresponding to 1.32% and 1.53% of the entire ZF1 genome, respectively. These archaic proportions are much higher than that in AK1 (Altai Neanderthal: 0.82%; Denisovan: 0.70%) or HX1 (Altai Neanderthal: 0.98%; Denisovan: 0.85%) (Fig. 4a). We further checked those novel archaic-shared sequences that could be unambiguously determined as an insertion model (Methods) and identified 239/164 Neanderthal/Denisovan-shared ZF1-specific events (sequences only present in ZF1 but absent in AK1 or HX1), which are more than the 133/115 Neanderthal/Denisovan-shared AK1-specific events and 151/126 Neanderthal/Denisovan-shared HX1-specific events, indicating the Tibetan genome contains more archaic-shared sequences than the other two East Asian genomes. After further filtering using the published European (NA12878) and African (NA19240) genomes, we obtained 167 Neanderthal- and 117 Denisovan-shared ZF1-specific events that were absent in the representative modern human assemblies (Methods, Fig. 4b and Supplementary Table 21), among which 51/28 Neanderthal/Denisovan-shared ZF1-specific events are present in great apes (chimpanzee, gorilla and orangutan).

Among the archaic-shared ZF1-specific NRSs, we found a 622-bp sequence in the intron of *SCUBE2* (Signal peptide-, CUB domain- and EGF-like domains-containing protein 2) (Fig. 4c), a non-repetitive insertion listed as the top three diverged insertions between Tibetans and Han Chinese (mV_ST_ = 0.079, Supplementary Fig. 12 and Supplementary Table 17). The 622-bp *SCUBE2* sequence is also present in great apes, suggesting that it is an ancestral sequence. We genotyped the *SCUBE2* insertion using PCR and Sanger sequencing in the three Tibetan populations (452 Lhasa samples, 214 Bange samples and 278 Langkazi samples), as well as in the Han Chinese population (92 samples) (Supplementary Fig. 17). The allele frequency of the *SCUBE2* insertion in Tibetans is on average near two-fold that in Han Chinese (0.240 vs 0.130 for the combined samples, Fig. 4d and Supplementary Table 22). The V_ST_ [CN] of this variant (0.096) is among the top 5% of the genome-wide SVs (5% cut-off = 0.0956) and is also significantly larger than the expected value under neutrality (see Methods, Supplementary Fig. 13).

Previous study found that *SCUBE2* could regulate VEGF-induced angiogenesis [38]. To check the functional relevance of the *SCUBE2* 662-bp insertion, we performed genetic association analysis in Tibetans (*n* = 944). We detected positive association with one lung index, the FEV1/FVC ratio (FFR) (FVC-forced vital capacity) (*P* = 0.04, FDR = 0.28) (Fig. 4e and Supplementary Table 19), although the association became non-significant after multiple-testing correction. In addition, using a joint additive model, we performed association analysis by combing the two SVs (the *MKL1* 163-bp deletion and the *SCUBE2* 662-bp insertion) and we observed a stronger signal for FFR compared with the single-SV analysis (*P* = 0.009, FDR = 0.060; Supplementary Fig. 18). It was known that Tibetans perform better than Han Chinese in view of lung functions at high altitude (i.e. larger FVC and FEV1) [23,39,40]. Collectively, these results suggest that the two SVs may work together to improve the lung function of Tibetans.

## Discussion

Through an integrated approach using PacBio long-read sequencing, BioNano optical mapping, 10X Genomics, Illumina HiSeq X10 and Hi-C technologies, we *de novo* assembled a high-quality Tibetan genome (ZF1). Compared with the previous *de novo* assemblies, the ZF1 assembly showed substantially improved quality with longer contig and scaffold N50 sizes. Based on this high-quality Tibetan genome, we detected 6505 ZF1-specific SVs and the associated genes are enriched for four functional clusters, especially for GTPase activity. Notably, GTPase activity is required for activation of hypoxia-inducible factor 1 (HIF-1α). In hypoxic cells, the small GTPase Rac1 is activated in response to hypoxia and is required for the induction of HIF-1α protein expression and transcriptional activity [41]. Consistently, the previously reported genes under selection in Tibetans (Supplementary Table 15) are enriched in the HIF-1 signaling pathway. Presumably, natural selection might have picked up some of these SVs contributing to high-altitude adaptation in Tibetans. Further population and functional data are needed to test the contribution of the GTPase-activity-related SVs to the regulation of the hypoxic pathway.

The identified SVs with base-level breakpoints accuracy offer a comprehensive map that could facilitate estimating the variant frequencies in the corresponding populations so that SVs with large divergence between highlander Tibetans and lowlander Han Chinese can be found. Importantly, combining TGS and NGS data, we successfully identified an intronic 163-bp deletion in *MKL1* and a 662-bp insertion in *SCUBE2* that are highly differentiated between Tibetans and Han Chinese. We speculate that these two SVs are likely under positive selection in Tibetans: (i) the considerable genetic differentiation between highlander Tibetans and lowlander Han Chinese is less likely caused by genetic drift or other demographic events under neutrality according to our simulation data; (ii) they show significant associations with multiple adaptive physiological traits in Tibetans and might have larger functional influence than SNPs in terms of nucleotide length. We noted that the two SVs did not show significant iHS or XP-EHH estimates over the genome, but this should not be the reason to rule out the possibility of positive selection on these loci. It is well acknowledged that different methods for detecting positive selection have their own underlying principles and weakness, and we should not expect positive results for a potential signal from all of them. In particular, most of the current methods were designed for SNP data and could have limited power when applied to SV analysis. The substantial genetic differentiation and phenotypic association suggest weak selection on the deletion at *MKL1* and the insertion at *SCUBE2*. Functional validation of these novel SVs would largely rely on the experimental studies in the future.

*MKL1* is a transcriptional regulator known to influence cellular response to stress signals in the vasculature. It was shown that, under chronic hypobaric hypoxia, the lung expression of *MKL1* was up-regulated in both rat and mouse, and *MKL1* knock-down could attenuate hypoxia-induced pulmonary hypertension (HPH) [34,35]. The *MKL1* protein directs histone H3 lysine 4 methyltransferase complexes to ameliorated HPH in mice [34]. Accordingly, the Tibetan-enriched *MKL1* 163-bp deletion is located in a putative enhancer sequence and embraced a GeneHancer regulatory element and two methylation hotspots (Fig. 3a and Supplementary Fig. 14), suggesting that it may affect epigenetic regulation of *MKL1* and eventually the downstream pathways, including vascular remodeling, vascular tone and pulmonary inflammation. Consistently, we saw negative association of the *MKL1* deletion with pulmonary arterial pressure in Tibetans, explaining their low hypoxic pulmonary vasoconstrictor response at high altitude [23]. In line with this view, based on SNV analysis, *MKL1* was recently reported to have undergone positive selection in the Himalayan populations from Nepal, Bhutan, North India and Tibet [33]. The 163-bp deletion may disrupt an enhancer of *MKL1*, leading to a reduced *MKL1* expression, subsequently attenuating CAM (cell adhesion molecules) and eventually ameliorating HPH (Supplementary Fig. 19) [34,35].

The high-quality genome allows us to better understand the sequences showing population-level or individual-level specificity where they are different or even absent from the human reference genome. ZF1 has more archaic-shared novel sequences than the other two Asians, consistently with a previous study proposing more archaic-shared DNAs in Tibetans than in Han Chinese [8]. The 662-bp *SCUBE2* insertion presents in both archaic and ZF1 genomes, but is absent in other Asian genomes. It is difficult to determine whether this insertion was introgressed from archaic hominids, but the association between the insertion and the lung-function index (the FEV1/FVC ratio) such as the 662-bp *SCUBE2* insertion (Supplementary Table 19) suggests that these archaic-shared sequences in modern Tibetans may contribute to high-altitude adaptation, in a way such as either selection acting on standing variants or like the reported ‘borrowed fitness’ case of *EPAS1* [22]. Of note, *SCUBE2* plays a key role for *VEGFR2* and potentiate VEGF-induced signaling in angiogenesis. *SCUBE2* is up-regulated by HIF1α at both mRNA and protein levels in lung endothelial cells [38], providing a possible mechanistic explanation for the observed association of the archaic-shared *SCUBE2* insertion with better lung functions in Tibetans.

Despite the success in charactering ZF1 SVs via analysing TGS data, there are some limitations in this study when using the NGS data to estimate the frequency of the SVs reported from ZF1. This is mainly due to the fact that near 70% of the SVs from TGS consist of repeat elements. We also found that the TGS-only deletions and insertions have different repeat proportions compared to the SVs that could be called by both the TGS and the NGS platform (P < 0.0001; Supplementary Fig. 20). The repeat elements would cause uncertainty for short-reads mapping and, in turn, affect the NGS SV detection. Consequently, the mismapped short reads would substantially influence the accuracy of SV detection. Given the uncertainty, we only considered those SVs with <70% of repeat elements and applied several stringent filtering steps to estimate the frequency difference between Tibetans and Han Chinese. This conservative strategy renders more accurate frequency estimation, while, on the other hand, it might miss highly differentiated variants, especially those containing a large portion of repeats. Such a problem could be solved in the future when the long-read sequencing becomes cost-effective for population studies.

In summary, taking advantage of long-read-sequencing and next-generation-mapping technologies, we *de novo* assembled a high-quality Tibetan genome and identified novel SVs, some of which might contribute to high-altitude adaptation in Tibetans. Our study demonstrates the value of constructing a high-resolution reference genome of representative populations (e.g. native highlanders) for understanding the genetic basis of human adaptation to extreme environments as well as for future clinical applications in hypoxia-related illness.

## METHODS

The detailed descriptions of methods are available as Supplementary Materials at *NSR* online.

## Data availability

The PacBio sequence data, Illumina sequencing reads, the ZF1 final assembly, the phased assemblies and its annotation files are available at the Genome Sequence Archive (GSA) (http://gsa.big.ac.cn/index.jsp) under the project ID of PRJCA000936. All data can also be viewed in NODE (http://www.biosino.org/node) by pasting the accession (OEP000207) into the text search box or through the URL http://www.biosino.org/node/project/detail/OEP000207.

## Supplementary Material

nwz160_Supplemental_File
