# Supplementary material for: De novo assembly of a Tibetan genome and identification of novel structural variants associated with high-altitude adaptation
Source: Natl Sci Rev. 2019 Oct 23;7(2):391–402. doi: 10.1093/nsr/nwz160 (PMC8288928; doi:10.1093/nsr/nwz160)
Supplement: nwz160_Supplemental_File [file nwz160_supplemental_file.pdf]

Supplementary Materials for

De novo assembly of a Tibetan genome and identification of novel structural variants associated with high altitude adaptation

Ouzhuluobu*, Yaoxi He*, Haiyi Lou*, Chaoying Cui*, Lian Deng*, Yang Gao, Wangshan Zheng, Yongbo Guo, Xiaoji Wang, Zhilin Ning, Jun Li, Bin Li, Caijuan Bai, Baimakangzhuo, Gonggalanzi, Dejiquzong, Bianba, Duojizhuoma, Shiming Liu, Tianyi Wu, Shuhua Xu†, Xuebin Qi†, Bing Su†

*These authors contributed equally to this work.
†Corresponding author Email: sub@mail.kiz.ac.cn (B.S.); qixuebin@mail.kiz.ac.cn (X.Q.); xushua@picb.ac.cn (S.X.)

This PDF file includes:
Methods
Supplementary Fig. 1-22
Supplementary Tables are provided as an Excel file.

Methods

ZF1 sample information

ZF1 is an adult male of native Tibetan ancestry, who has lived in Lhasa (3,680m) for more than 30 years. He is healthy (measured by physical exam and self-report), normotensive, non-anemic, normal pulmonary function and nonsmoking (by self-report). Written informed consent was obtained from ZF1. Freshly drawn blood samples were collected for DNA extraction. The protocol of this study was reviewed and approved by the Internal Review Board of Kunming Institute of Zoology, Chinese Academy of Sciences (Approval ID: SWYX-2012008) and Tibetan University (Approval ID: 2011-XZDX-001).

Data Generation

PacBio data: high-quality genomic DNA was extracted from blood sample using the Phenol-Chloroform method and sequenced by the PacBio sequencer RSII (P6-P4 sequencing reagent).

BioNano data: according to the protocol provided by Bionano Genomics company, we obtained the high-molecular-weight DNA and constructed the high-quality sequencing library. Nt.BspQI was used for enzyme digestion. We used Irys system to analyze Bionano data.

10X Genomics data: high-molecular-weight DNA was used to construct the DNA library, and the protocol from 10X Genomic Chromium™ and Illumina Hiseq sequencer was adopted to generate long linked-reads data.

Hi-C data: adequate lymphocytes (1.5×10^7) were extracted from fresh human peripheral blood. We constructed library by the previously published protocol and performed Illumina HiSeq PE150 sequencing to generate Hi-C data.

HiSeq XTen data: the 100× paired-end reads (150-bp) were generated using Illumina HiSeq X sequencer.

De novo assembly

We obtained in total 24,880,404 subreads from PacBio RSII sequencer, and all these long reads were error-corrected and assembled into contigs using Falcon (v3.0) (https://github.com/PacificBiosciences/FALCON-integrate), and then polished by Quiver [1]. For scaffolding the contigs, we adopted two different strategies to enhance the assembling:

Strategy-1: after obtaining the assembled contigs and error-corrected long-reads, we mapped 10X Genomics linked-reads with these contigs and anchored them into preliminary scaffolds. We then generated the optical maps and the Irys BioNano platforms (Irys System, BioNano Genomics) was used in scaffolding preliminary scaffolds to elongations by the hybrid scaffold pipeline (Bionano Solve v3.1) (Supplementary Fig. 2a and Supplementary Table 2).

Strategy-2: with the contigs assembled from PacBio reads, we first hybrid-assembled them into preliminary scaffolds using BioNano optical map using hybrid scaffold pipeline (same as above). Then we mapped 10X Genomics linked-reads with the preliminary scaffolds based on the supported linked-reads (Supplementary Fig. 2b and Supplementary Table 3).

Next, we aligned the Hi-C reads using BWA [2] with default parameters. Long scaffolds within each chromosomal linkage group were then assigned based on the Hi-C-based proximity-guided assembly. The original cross-linked long-distance physical interactions were then processed into paired-end sequencing libraries. First, all the reads from the Hi-C libraries were filtered by the HiC-Pro software (v2.8.1) [3], and the paired-end reads were uniquely mapped onto the draft assembly scaffolds, which were then grouped into 24 chromosome clusters using SALSA software [4] (Supplementary Table 23 and 24). The clustering errors were corrected by referring to GRCh38.

PBJelly v.15.8.24 [5] was used to close gaps of draft genomes. Briefly, all the gaps (length ≥25 bp) on the assembly were identified. Then, the long Pacbio reads were aligned to the scaffold genome using PBJelly. After read alignment, the supporting procedure was parsed by checking the multi-mapping information. After the gap-supporting sequence reads are identified, PBJelly assembles the reads for each gap to generate a high-quality gap-filling consensus sequence. Finally, the assembly was

polished with Illumina reads by aligning the paired-end short-reads to the assembly using BWA. Picard was used to remove duplications within reads, and base-correction of the assembly was performed using Pilon [6] (Supplementary Fig. 2).

Phasing the diploid assembly

We used HapCUT2 [7] and CrossStitch (https://github.com/schatzlab/crossstitch) to generate a phased genome with all the variants (SVs from PacBio, SNVs and INDELs from 10X Genomics).

Gap closure in the human reference genome GRCh38

We closed the gaps in the human reference genome (GRCh38) by using the approach of the previous study [8]. A region consisting of continuous runs of Ns in the GRCh38 was defined as a gap. We extracted these GRCh38 gaps based on the BED file format, and the 5kb flanking sequences upstream and downstream of the gaps were mapped to the assembly by MUMmer (*nucmer -f -r -l 15 -c 25*). A gap is defined as closed only if the two flanking sequences in GRCh38 could both be aligned to the ZF1 assembly with consistent orientation, and the aligned length is over 2.5kb. The added bases were precisely counted according to the position of the two flanking sequences on the ZF1 assembly.

Evaluation of consensus quality and sequence quality

Consensus quality of the ZF1 assembly was evaluated by comparing each chromosome with the reference genome GRCh38 using MUMmer [9] (arguments: *nucmer --mum -c 1000 -l 100; delta-filter -i 85 -l 1000 -1*).

We mapped all 100× Illumina short reads to the ZF1 assembly using the BWA-MEM [2] module. Then we used *Picard* to mask the PCR duplicates and generated the dedup.bam file. Variants were called by the Haplotype Caller module of Genome Analysis Toolkit (GATKv3.6) (https://www.broadinstitute.org/gatk/) [10]. The SNPs and INDELs were filtered using the GATK Variant Filtration module with the following criteria, respectively: SNPs filtering: "QUAL <50; QD < 2.0; FS > 60.0; MQ < 30.0;

MQRankSum < -12.5; ReadPosRankSum < -8.0; DP < 30"; INDELs filtering: "QUAL <50; QD < 2.0; FS > 200.0; ReadPosRankSum < -20.0; DP < 30". As the previous studies described [11, 12], we counted the total number of the homozygous SNVs (SNPs+INDELs) which represent the sites with base errors in the ZF1 assembly. The base-error rate was calculated as the number of homozygous sites divided by the total sites of the ZF1 assembly (Supplementary Table 6).

Gene Annotation

We used GRCh38 (http://ftp.ensemblorg.ebi.ac.uk/pub/release-90/fasta/homo_sapiens/dna/) as the reference annotation panel. After performing repeat masking to ZF1 and the reference panel, we aligned ZF1 to GRCh38 by Last [13] and generated the maf file. Then we applied CESAR2.0 (Coding Exon Structure Aware Realigner 2.0, http://github.com/hillerlab/CESAR2.0) [14] to identify genes and the coding exons. Functional annotation for the ZF1 genes was performed using four databases: KEGG (https://www.genome.jp/kegg/), Swiss-Prot (https://www.uniprot.org/), InterPro (http://www.ebi.ac.uk/interpro/) and NR (https://www.ncbi.nlm.nih.gov/refseq/) (Supplementary Fig. 5).

Detection of SVs

For long-read PacBio data, we used mapping software NGLMR [15] to align the error-corrected reads ('preads') from Falcon output to the human reference genome GRCh37. We used GRCh37 instead of GRCh38 for SV detection because the majority of the previously reported SVs were based on GRCh37, and the downstream analyses included the comparisons of these SVs among different populations. Then we used Sniffles [15] to call SVs from the bam file and we required each variant with support from at least ten reads. For the NGS short-read Illumina data, we mapped the reads to the human reference genome GRCh37 using BWA. After sorting and removing duplicates, we used CNVnator [16], Pindel [17], Lumpy [18], BreakDancer [19] and BreakSeq2 [20] to call SVs. We further merged the results from five algorithms by MetaSV [21]. We also used PopIns [22] to call the non-reference non-repetitive

insertions from Illumina data. For BioNano data, we detected SVs by Irys Solve (v3.1). For the 10X Genomics data, Long Ranger (v2.1.6) was applied to call genetic variants including SVs, SNVs and INDELs. The SNVs and INDELs were used in the phasing analysis.

Estimation of SV mutation rate

We used the Watterson's θ to estimate the mutation rate, which is calculated as below:

$$\theta = \frac{K}{\sum_{i}^{n-1} \frac{1}{i}}$$

where K is the total number of segregating SV sites and n is the number of haploid genomes. Then we assumed the effective population size Ne=10,000[23] and calculated the mutation rate μ as below:

$$\theta = 4Ne\mu$$

SV annotation and enrichment analysis

Annotation for ZF1 SVs were defined by VEP (http://www.ensembl.org/info/docs/tools/vep/index.html) [24]. Repeat analysis for SVs region were used by RepeatMasker v4.0.1. Function enrichment analysis was perform by DAVID v6.7 [25]. In addition, we calculated the odds ratio to evaluate the enrichment of the genes affected by SVs in a set of priori candidate genes (the hypoxia regulatory genes or previously reported adaptive genes in Tibetans).

$$\text{Odds Ratio} = \frac{S_1}{S_0} \Big/ \frac{N_1}{N_0}$$

where S_1 denotes the number of SV genes presenting in the priori candidate gene list; S_0 denotes the number of SV genes absent from the priori candidate gene list; N_1 denotes the number of non-SV genes presenting in the priori candidate gene list; N_0 denotes the number of non-SV genes absent from the priori candidate gene list. The sum of S_1, S_0, N_1 and N_0 is the total number of genes across the genome. An odds ratio significantly above 1 ($p < 0.05$, the Chi-squared test) indicates that the SV genes are enriched in the priori gene set.

Overlap enrichment analysis of SVs versus genomic elements

We performed permutation tests for functional genomic elements overlapped with four different class of SVs (DEL, DUP, INS and INV). The genomic elements used in this study were from previous study [23]. The null distribution (random background) of the overlap counts was calculated from the true genomic elements overlapped with the random shuffled SV locations. We shuffled each type of SV for 1,000 times, and generated 1,000 random SV sets. The same number of SVs and the same length distribution of SVs of each SV type was kept in each shuffled set. We adopted log2 fold change of the observed overlap statistic versus the mean of the null distribution to present the enrichment of genomic element-SV overlap.

SV genotype estimation using NGS data

We used the deletions and duplications detected by long-read sequencing platform as candidate copy number variable regions to further investigate the frequency difference in Tibetan and Han Chinese populations. The population genomic data are whole-genome sequenced (~30X) from a previous study (38 Tibetans and 39 Han Chinese) using Illumina HiSeq X10 [26]. As the short-read NGS data have bias for reads coverage at certain genomic regions [27], we applied a stringent strategy to filter the CNV regions where we could get high quality results from NGS data. First, we filtered out the TGS CNVs where the variants could not be detected from ZF1 NGS data. Then we used CNVnator to obtain the genotype for each remaining CNV region in ZF1 sample and removed the region where the NGS genotype was inconsistent with TGS calling (consistent NGS genotype range: CNVnator genotype<1.3 for TGS deletion; 2.7<CNVnator genotype<4.3 for TGS duplication; we excluded the regions with copy number>4 due to the inaccurate estimation of high copy number variants from NGS). Next, we genotyped the remaining CNV regions for each of the 38 Tibetan and 39 Han Chinese samples using CNVnator. Based on the rounding results of the CNV genotypes, we removed the CNV regions that failed to pass the parity test [28] in either Tibetan or Han Chinese population. V_{ST} [29] was used to measure allelic divergence between Tibetans and Han Chinese at the 1,887 CNV regions which passed all the filtering steps above.

To obtain population frequency of non-repetitive insertions, we used the assembled ZF1 genome (including the associated contigs) as reference and aligned short-reads of 38 Tibetan and 39 Han Chinese NGS data to this reference. For each insertion with sequence available reported by Sniffles, we located the positions of these sequences on the ZF1 assembly and removed the duplications using Lastz [30] (--*notransition --nogapped –step=20 –filter=identity:90 –filter=coverage:90*); we excluded insertions with more than 70% of repeats reported by Tandem Repeat Finder (TRF) or RepeatMasker. Next we determined the copy number (CN, e.g. 0, 1, 2..) of insertions for each sample by rounding the value of two times of the relative read-depth of the insertion. The relative read-depth was calculated as the average read depth of inserted sequence divided by the average whole genome coverage. The average read depth was calculated using SAMtools depth module. A total of 593 non-repetitive insertions were included in the analysis. Finally, to mitigate the potential batch effects from NGS data, we used a conservative way to measure the insertion frequency differentiation between Tibetan and Han Chinese by taking the minimum (mV_{ST}) of the two V_{ST} values for each insertion locus: one was directly based on CN states (V_{ST}[CN], as calculated for the CNV differentiation), and the other one (V_{ST}[norm-RD]) was based on median-normalized relative read-depth (that is, the relative read-depth divided by the median in each population; if the median equals to zero, then no normalization was performed).

We listed the top 5% of the V_{ST} and mV_{ST} for CNVs and insertions (calculated separately) in Supplementary Table 16 and 17 respectively. The 5% of the empirical V_{ST} (V_{ST}[CN]) is 0.0956 corresponding to the 98.3 percentile in the simulated null distribution (see section below).

Simulation of SV frequency differentiation between Tibetans and Han Chinese

We employed *ms* [31] to generate a null V_{ST} distribution to assess the SV frequency differentiation between Tibetans and Han Chinese under neutral evolution. Following previous studies [26], we assumed that Tibetans and Han Chinese split 10,000 years ago (*T3*), and after the divergence, a bottleneck event in Tibetans occurred till 9,000

years before present (*T2*). We also considered an exponential growth of effective population size (*Ne*) for Han Chinese starting at 2,000 years before present (*T1*). We assumed the *Ne* of the Tibetan-Han Chinese common ancestry (*N1*) to be 20,000, the *Ne* at *T2* in Tibetan to be 5,000 (*N2*), and the *Ne* for present Tibetans and Han Chinese at *T3* to be 20,000 (*N3*) and 50,000 (*N4*) respectively (Supplementary Fig. 21). We assumed generation time of 25 years and the mutation rate of SV to be 10^{-5} per generation [32]. The following *ms* command was used to perform the simulation:

ms 154 2000 -t 0.4 -s 1 -I 2 78 76 -g 1 458.1 -n 1 5 -n 2 2 -eg 0.002 1 0 -en 0.009 2 0.5 -ej 0.01 2 1

SV validation using PCR and Sanger sequencing

We genotyped the candidate SVs in ~900 unrelated Tibetans and ~100 unrelated Han Chinese samples using PCR and Sanger sequencing. The primers were designed by Primer Premier 5, the extended 200bp sequences were included at SV breakpoints as PCR target and sequenced using Sanger sequencing.

Physiological traits measurement and association analysis of Tibetan populations

We collected physiological traits data and blood samples from 1,039 Tibetan volunteers who are native residents at the sampled locations, and they were from three different altitude regions in Tibet, including Lhasa (elevation: 3,658m), Bange (elevation: 4,700m) and Langkazi (elevation: 5,108m). We also sampled a Han Chinese population (n=100) from Dalian, China (elevation: 60m) as the reference. We filtered the samples based on the following criteria: 1) healthy (by physical examination and self-report); 2) normotensive, non-anemic, normal pulmonary function and non-pregnant; 3) $18 \leq age \leq 70$; 4) nonsmoking (by self-report). The ethnic identity was confirmed by self-claims and by report of the first language learned, and related individuals were excluded. Written informed consents were obtained from all participants.

We collected venous blood (5 ml from each individual) from subjects who fasted overnight. We measured a total of 19 physiological traits including NO: serum nitric oxide level; PAP: systolic pulmonary arterial pressure; SPO2: peripheral capillary

oxygen saturation. HB: hemoglobin concentration, RBC: red blood count; HCT: hematocrit; MCV: mean red cell volume; RDW: red cell distribution width; PLT: platelets; LPC: lymphocyte count; SBP: systolic blood pressure; DBP: diastolic blood pressure; HR: heart rate; PEF: peak expiratory flow rate (L/min); MVV: Maximum Ventilatory Volume (L/min); FEF: Forced expiratory flow at 25%-75% (L/min); FEV1: Forced Expiratory Volume In 1s (%); FFR: FEV1/FVC; FVC: forced vital capacity (L). The HB concentration and other blood parameters were measured immediately using an automated hematology analyzer (Sysmex pocH-100i, Japan). SPO2 was measured at forefinger tip with a hand-held pulse oximeter (Nellcor NPB-40, CA) at rest, and the fingertip was cleaned with alcohol swab before measurement. Serum NO levels were measured by protocol as we described before [33]. For lung function test, we performed on a Microlab Spirometer 3500K, version 5.X.X Carefusion (Micro Medical Ltd., Rochester, United Kingdom) according to the ATS (American thoracic society) recommendation and the international standardized guideline [34]. Subjects were kept sitting position with a nose clip, and after two or three slow vital capacity tests, we collected the results of at least three forced vital capacities. The highest of the recorded FVC values was reported in the present study. PEF, MVV, FEF and FEV1 were measured as primary data. Stringent quality control was conducted in the entire procedure.

Genetic association analysis of physiological traits was performed using PLINK 1.07 [35]. We used additive model to evaluate the association between SVs and phenotypes. Sex, age and altitude were treated as covariates for association analysis of all phenotypes. For lung functions (PEF, MVV, FEF, FEV1, FVC and FFR), we also took BMI as the covariate. To test the joint effect on association of the *MKL1*-163bp-deletion and the *SCUBE2*-662bp-insertion, we employed a joint-additive model and took the allelic status of the *MKL1* 163bp-deletion as the covariate (by plink: "—*condition*" argument). For multiple test correction, we used Benjamini & Hochberg (1995) step-up FDR control to adjust the P values using R function of *p.adjust*. We performed association test for samples from Lhasa, Bange and Langkazi separately. We

performed the heterozygosity test including Cochrane's Q statistics and I^2 heterogeneity index (Q>0.1 and $I^2<25$ for homogeneous) [36], and found no genetic heterogeneity before pooling together the three populations.

Non-reference sequences shared by archaic hominid and de novo assembled Asian genomes

To search for the sequences that are present in the Asian genomes (*i.e.* AK1, HX1 or ZF1) and the archaic hominids (Neanderthal or Denisovan) but absent in the human reference genome, we aligned the archaic short reads that could not be mapped to GRCh37 (reference-unmapped reads, RURs) to each of the individual genomes. We used the high-coverage Altai Neanderthal genome (~51×) from reference [37], and the RURs were downloaded from (http://cdna.eva.mpg.de/neandertal/altai/AltaiNeandertal/bam/unmapped_qualfail/). The high-coverage Denisovan sequencing data (~30×) were from the literature [38], and we processed the Denisovan raw reads following the published protocols [38] to align to human reference GRCh37 and extracted the RURs. Then we mapped the Neanderthal and Denisovan RURs to each of the individual genomes (*i.e.* AK1, HX1 or ZF1 contigs) using *bwa aln* [2]. We treated all the archaic pair-end RURs as single-end reads during mapping. After mapping, we considered the regions with archaic RURs reaching an average depth with range between 1/3 and 1.5 folds of the genome-wide depth of the archaic reads mapped to reference human GRCh37 (*i.e.* Neanderthal: (17, 75), Denisovan: (10, 45)), as such depth range indicates that the archaic genome might likely contain one or two copies of these sequences. We further used Lastz (*--notransition --nogapped –step=20 –filter=identity:90 –filter=coverage:90*) [30] to align the sequences of these regions to GRCh37 and removed the sequences with high-similarity in the human reference genome. The proportion of each individual genome sharing the novel sequences with archaic hominids was calculated as the total region length with the depth falling the range above divided by the total size of the individual genome.

To obtain the positions of these sequences regarding the human reference genome,

we aligned individual contigs (ZF1, HX1 and AK1) with GRCh37 using MUMmer [9], and we only considered the sequences where their flanking positions could be determined based on GRCh37 coordinates. As shown in Supplementary Fig. 22, we required both the aligned segments larger than 500-bp (c>500 & d>500) and filtered out the contigs with less than 50% coverage of alignments (a/(c+d)<0.5). We required the gap between two alignments on ZF1 contig to be larger than that on human reference genome (a>b). The region with archaic reads mapping must contain more than five reads, and the region length must be greater than half of the gap between two alignments on ZF1 contig (a'>a/2). The inserted sequences meeting all the above conditions were considered as novel sequences with clear positions on the human reference genome. As the sub-telomeric and sub-centromeric regions contain lots of repeats, we further removed the sequences located within 1Mb of telomeres or centromeres. We focused on the sequence that the archaic RUR could only align to one of the three Asian individual contigs but not the other two individual contigs. Such sequences were referred as individual-specific novel sequences shared with archaic hominids. In order to check whether these individual-specific novel sequences could be found in other modern humans other than East Asians, by using Lastz (*--notransition --nogapped –step=20 –filter=identity:95 –filter=coverage:95*), we further aligned the ZF1-specific sequences to two additional modern human *de novo* assemblies (NA12878 and NA19240 downloaded from NCBI with accession PRJNA323611) [12], which represent European and African genomes respectively. To assess whether the ZF1-specific sequences present in non-human primates, using Lastz (*--notransition --nogapped –step=20 –filter=identity:80 –filter=coverage:80*), we aligned the sequences to the genomes of chimpanzee, gorilla and orangutan [12].

Estimation of EHH

To examine whether there is a selective sweep around the *SCUBE2* 622-bp insertion (Chr11: 9068607) and the *MKL1* 163-bp deletion (Chr22:40935468-40935631), we first phased the genotypes in the 1-Mb region around these variants (Chr11: 8568607-9568607 and Chr22:40435468-41435631), respectively, in the combined dataset of the

whole-genome sequences of 39 Tibetan highlanders and 38 Han Chinese lowlanders, using SHAPEIT v2 [39] (r837) without any reference populations. We then calculated the EHH statistics for the focal SVs compared to the alternative alleles in these two populations, and the result was visualized using an R package *rehh* [40] with default parameters. The ancestral allele for each locus was determined according to the ancestral sequences released by the 1000 Genomes Project.

Reference

1. Chin CS, Alexander DH, Marks P*, et al.* Nonhybrid, finished microbial genome assemblies from long-read SMRT sequencing data. *Nature Methods*. 2013; **10**: 563-+.
2. Li H, Durbin R. Fast and accurate long-read alignment with Burrows-Wheeler transform. *Bioinformatics*. 2010; **26**: 589-95.
3. Servant N, Varoquaux N, Lajoie BR*, et al.* HiC-Pro: an optimized and flexible pipeline for Hi-C data processing. *Genome Biology*. 2015; **16**: 11.
4. Ghurye J, Pop M, Koren S*, et al.* Scaffolding of long read assemblies using long range contact information. *Bmc Genomics*. 2017; **18**.
5. English AC, Richards S, Han Y*, et al.* Mind the Gap: Upgrading Genomes with Pacific Biosciences RS Long-Read Sequencing Technology. *Plos One*. 2012; **7**.
6. Walker BJ, Abeel T, Shea T*, et al.* Pilon: An Integrated Tool for Comprehensive Microbial Variant Detection and Genome Assembly Improvement. *Plos One*. 2014; **9**.
7. Edge P, Bafna V, Bansal V. HapCUT2: robust and accurate haplotype assembly for diverse sequencing technologies. *Genome Res*. 2017; **27**: 801-12.
8. Shi LL, Guo YF, Dong CL*, et al.* Long-read sequencing and de novo assembly of a Chinese genome. *Nature Communications*. 2016; **7**.
9. Kurtz S, Phillippy A, Delcher AL*, et al.* Versatile and open software for comparing large genomes. *Genome Biology*. 2004; **5**.
10. McKenna A, Hanna M, Banks E*, et al.* The Genome Analysis Toolkit: A MapReduce framework for analyzing next-generation DNA sequencing data. *Genome Research*. 2010; **20**: 1297-303.
11. Du H, Yu Y, Ma Y*, et al.* Sequencing and de novo assembly of a near complete indica rice genome. *Nature Communications*. 2017; **8**.
12. Kronenberg ZN, Fiddes IT, Gordon D*, et al.* High-resolution comparative analysis of great ape genomes. *Science*. 2018; **360**: 1085-+.
13. Kielbasa SM, Wan R, Sato K*, et al.* Adaptive seeds tame genomic sequence comparison. *Genome Research*. 2011; **21**: 487-93.
14. Sharma V, Schwede P, Hiller M. CESAR 2.0 substantially improves speed and accuracy of comparative gene annotation. *Bioinformatics*. 2017; **33**: 3985-7.
15. Sedlazeck FJ, Rescheneder P, Smolka M*, et al.* Accurate detection of complex structural variations using single-molecule sequencing. *Nature Methods*. 2018; **15**: 461-+.
16. Abyzov A, Urban AE, Snyder M*, et al.* CNVnator: An approach to discover, genotype, and characterize typical and atypical CNVs from family and population genome sequencing. *Genome*

Research. 2011; **21**: 974-84.
17. Ye K, Schulz MH, Long Q*, et al.* Pindel: a pattern growth approach to detect break points of large deletions and medium sized insertions from paired-end short reads. *Bioinformatics*. 2009; **25**: 2865-71.
18. Layer RM, Chiang C, Quinlan AR*, et al.* LUMPY: a probabilistic framework for structural variant discovery. *Genome Biology*. 2014; **15**.
19. Chen K, Wallis JW, McLellan MD*, et al.* BreakDancer: an algorithm for high-resolution mapping of genomic structural variation. *Nature Methods*. 2009; **6**: 677-U76.
20. Abyzov A, Li ST, Kim DR*, et al.* Analysis of deletion breakpoints from 1,092 humans reveals details of mutation mechanisms (vol 6, 7256, 2015). *Nature Communications*. 2015; **6**.
21. Mohiyuddin M, Mu JC, Li J*, et al.* MetaSV: an accurate and integrative structural-variant caller for next generation sequencing. *Bioinformatics*. 2015; **31**: 2741-4.
22. Kehr B, Helgadottir A, Melsted P*, et al.* Diversity in non-repetitive human sequences not found in the reference genome. *Nat Genet*. 2017; **49**: 588-93.
23. Sudmant PH, Rausch T, Gardner EJ*, et al.* An integrated map of structural variation in 2,504 human genomes. *Nature*. 2015; **526**: 75-81.
24. McLaren W, Gil L, Hunt SE*, et al.* The Ensembl Variant Effect Predictor. *Genome Biology*. 2016; **17**.
25. Dennis G, Sherman BT, Hosack DA*, et al.* DAVID: Database for annotation, visualization, and integrated discovery. *Genome Biology*. 2003; **4**.
26. Lu DS, Lou HY, Yuan K*, et al.* Ancestral Origins and Genetic History of Tibetan Highlanders. *American Journal of Human Genetics*. 2016; **99**: 580-94.
27. Benjamini Y, Speed TP. Summarizing and correcting the GC content bias in high-throughput sequencing. *Nucleic Acids Research*. 2012; **40**.
28. Handsaker RE, Van Doren V, Berman JR*, et al.* Large multiallelic copy number variations in humans. *Nature Genetics*. 2015; **47**: 296-+.
29. Redon R, Ishikawa S, Fitch KR*, et al.* Global variation in copy number in the human genome. *Nature*. 2006; **444**: 444-54.
30. Harris RS. IMPROVED PAIRWISE ALIGNMENT OF GENOMIC DNA *Doctor of Philosophy*. The Pennsylvania State University 2007.
31. Hudson RR. Generating samples under a Wright-Fisher neutral model of genetic variation. *Bioinformatics*. 2002; **18**: 337-8.
32. Campbell CD, Eichler EE. Properties and rates of germline mutations in humans. *Trends in Genetics*. 2013; **29**: 575-84.
33. He YX, Qi XB, Ouzhuluobu*, et al.* Blunted nitric oxide regulation in Tibetans under high-altitude hypoxia. *Natl Sci Rev*. 2018: 1-14.
34. Miller MR, Hankinson J, Brusasco V*, et al.* Standardisation of spirometry. *European Respiratory Journal*. 2005; **26**: 319-38.
35. Purcell S, Neale B, Todd-Brown K*, et al.* PLINK: a tool set for whole-genome association and population-based linkage analyses. *Am J Hum Genet*. 2007; **81**: 559-75.
36. Cochran WG. THE COMPARISON OF PERCENTAGES IN MATCHED SAMPLES. *Biometrika*. 1950; **37**: 256-66.
37. Prufer K, Racimo F, Patterson N*, et al.* The complete genome sequence of a Neanderthal from the Altai Mountains. *Nature*. 2014; **505**: 43-+.
38. Meyer M, Kircher M, Gansauge MT*, et al.* A High-Coverage Genome Sequence from an Archaic

Denisovan Individual. *Science*. 2012; **338**: 222-6.
39. Delaneau O, Marchini J, Zagury J-F. A linear complexity phasing method for thousands of genomes. *Nature Methods*. 2012; **9**: 179-81.
40. Gautier M, Vitalis R. rehh: an R package to detect footprints of selection in genome-wide SNP data from haplotype structure. *Bioinformatics*. 2012; **28**: 1176-7.
41. Chen DW, Yang YY, Cheng X*, et al.* Megakaryocytic Leukemia 1 Directs a Histone H3 Lysine 4 Methyltransferase Complex to Regulate Hypoxic Pulmonary Hypertension. *Hypertension*. 2015; **65**: 821-+.
42. Yuan ZB, Chen J, Chen DW*, et al.* Megakaryocytic Leukemia 1 (MKL1) Regulates Hypoxia Induced Pulmonary Hypertension in Rats. *Plos One*. 2014; **9**.

Supplementary Figures

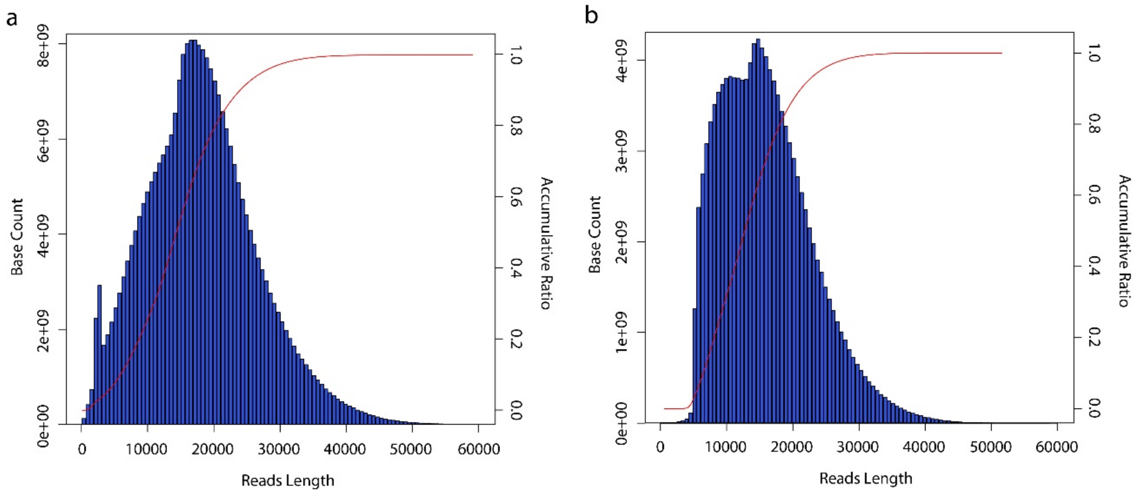

Supplementary Fig. 1 | Length distribution of raw reads and error-corrected subreads. a. Read length distribution of raw reads. b. Read length distribution of error-corrected subreads, which are used for de novo assembly and SV detection.

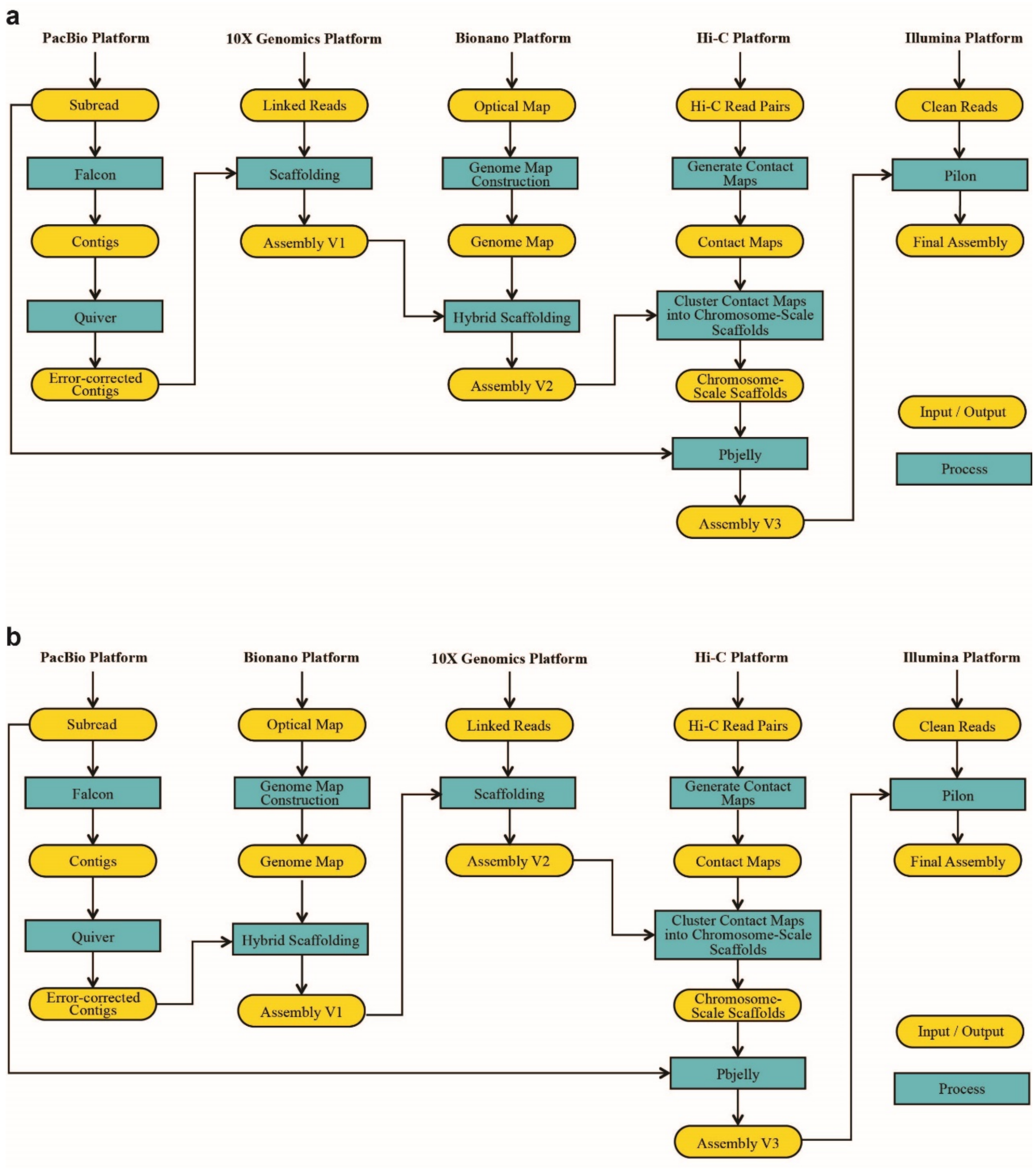

Supplementary Fig. 2 | Overview of data generation and *de novo* assembly pipeline. Two different versions of scaffolding are shown as a. and b., respectively.

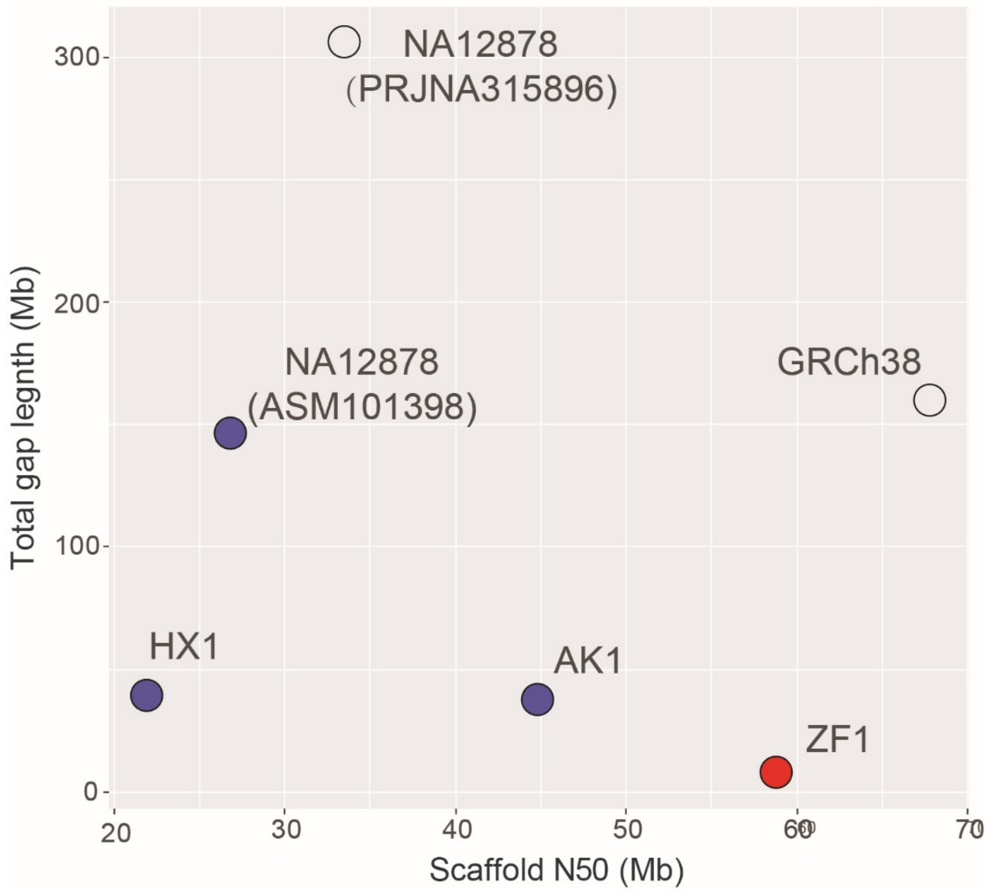

Supplementary Fig. 3 | Comparison between the ZF1 assembly and five previous high-quality assemblies. The solid circles refer to the genome assembles by PacBio long reads, and the hollow circles refer to the genome assembles by other sequencing data without PacBio long reads.

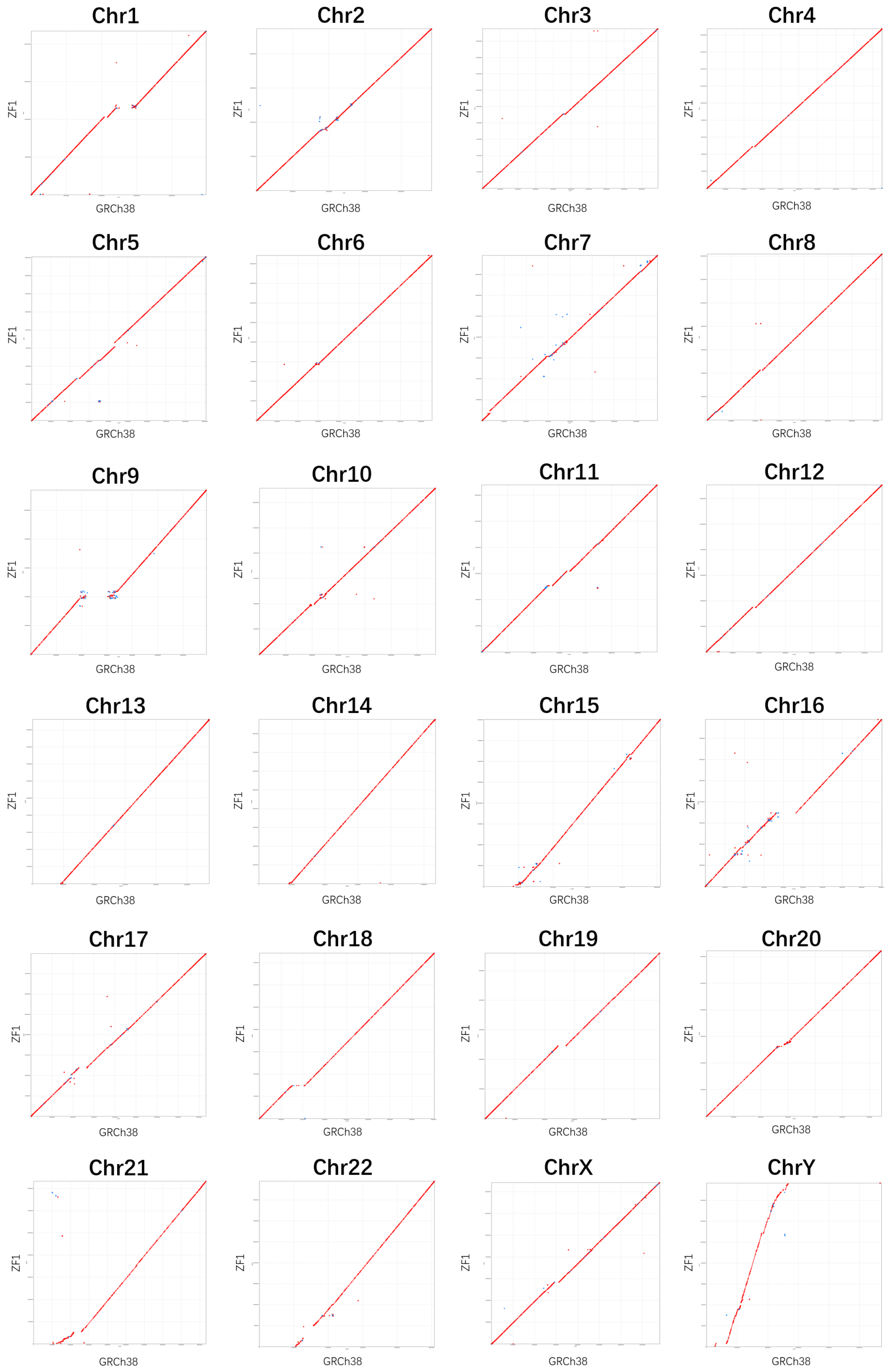

Supplementary Fig. 4 | Dot plots of comparison between ZF1 assembly and GRCh38 assembly.

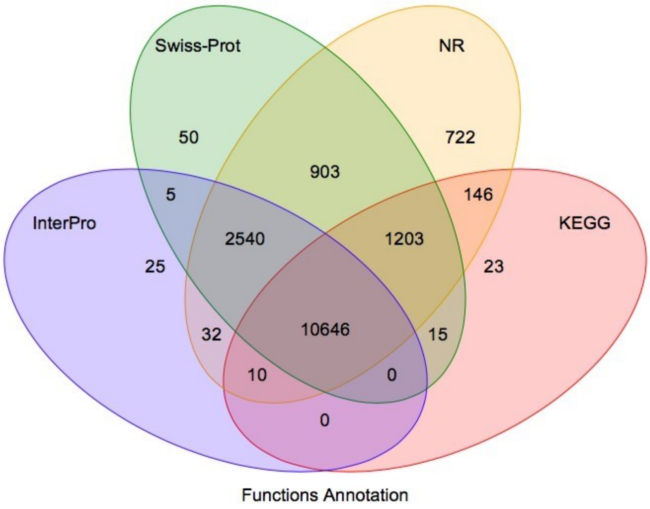

Supplementary Fig. 5 | Summary of function annotation of ZF1 by different databases.

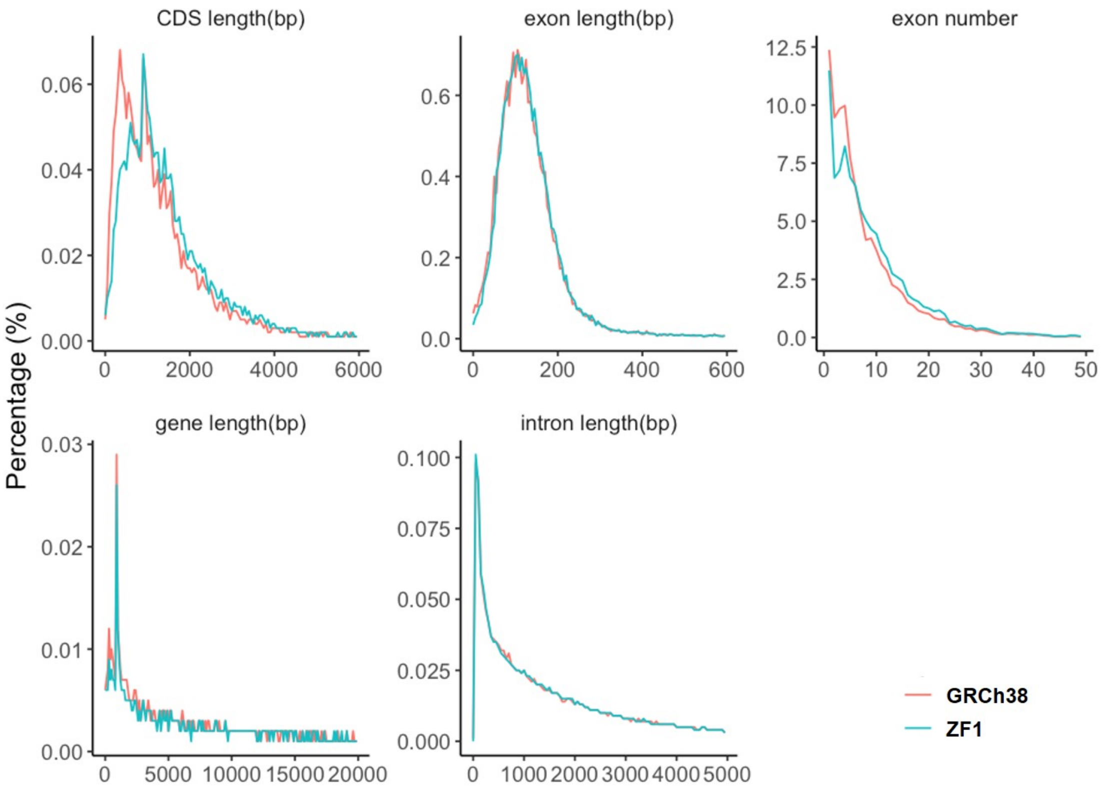

Supplementary Fig. 6 | Comparison of function elements between GRCh38 and ZF1

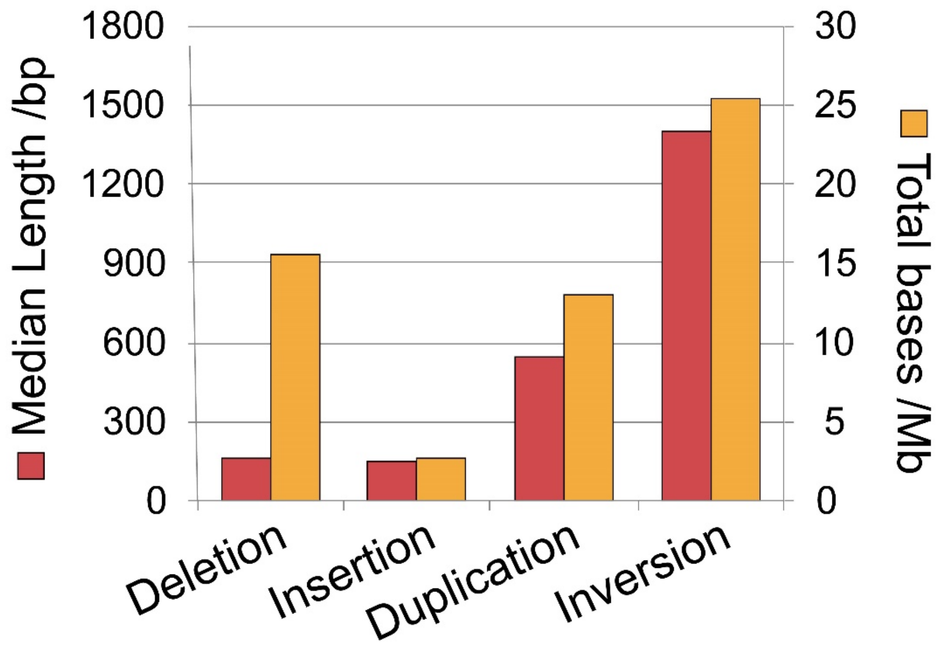

Supplementary Fig. 7 | Median length and total base statistics of different SVs.

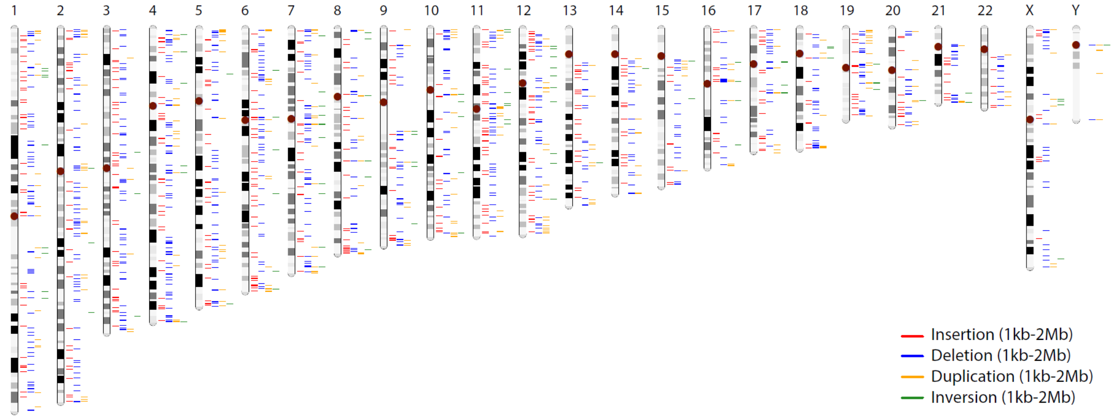

Supplementary Fig. 8 | Genome-wide distribution of large-scale structure variants of ZF1 (1kb-2Mb)

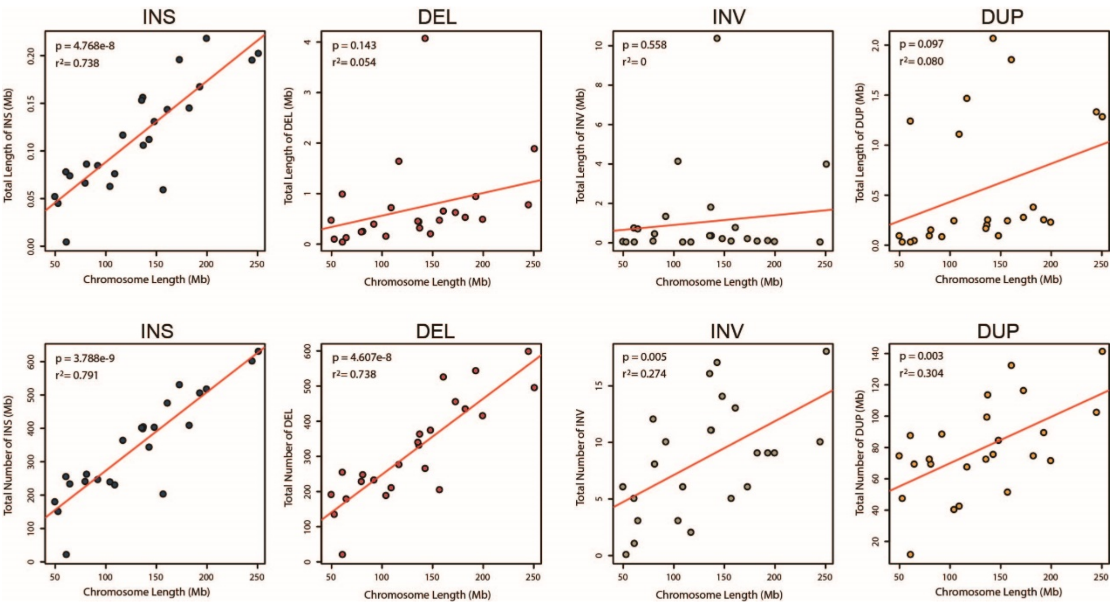

Supplementary Fig. 9 | Correlation with each chromosome length and SV numbers and SV length.

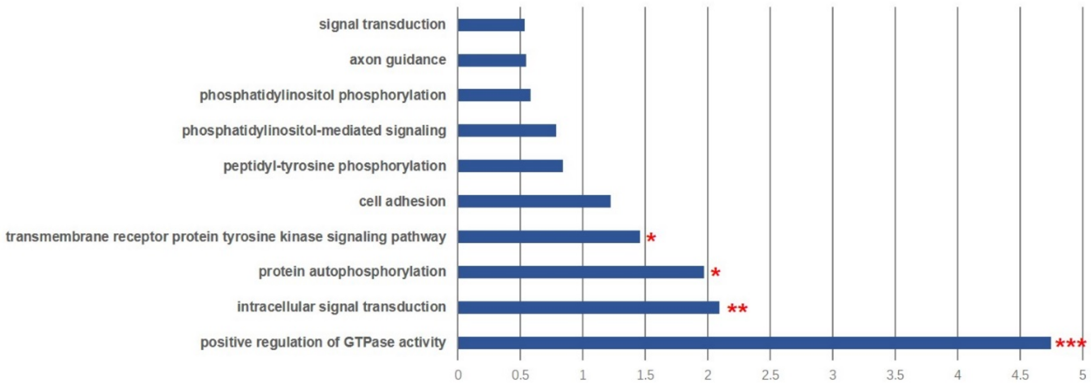

Supplementary Fig. 10 | Functional enrichment of the ZF1-specific SVs. The significant terms were marked “*”.

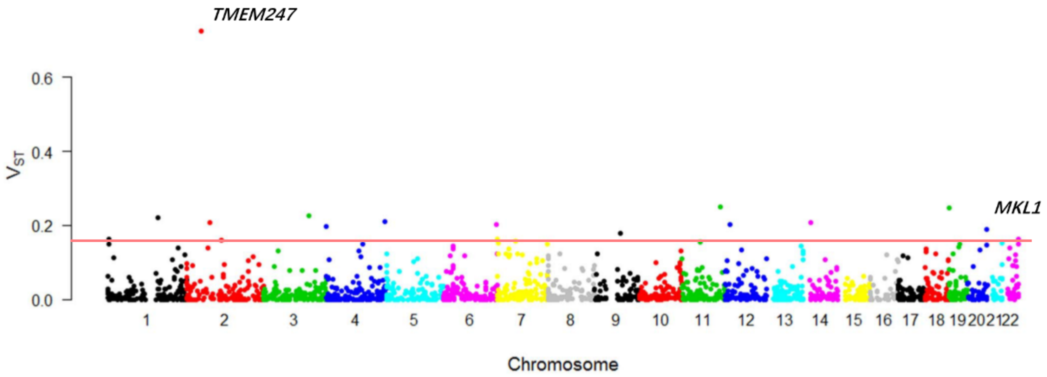

Supplementary Fig. 11 | Manhattan plot of V_{ST} between Tibetan and Han Chinese. The red line refers to the top 5% of 1887 candidate CNVs.

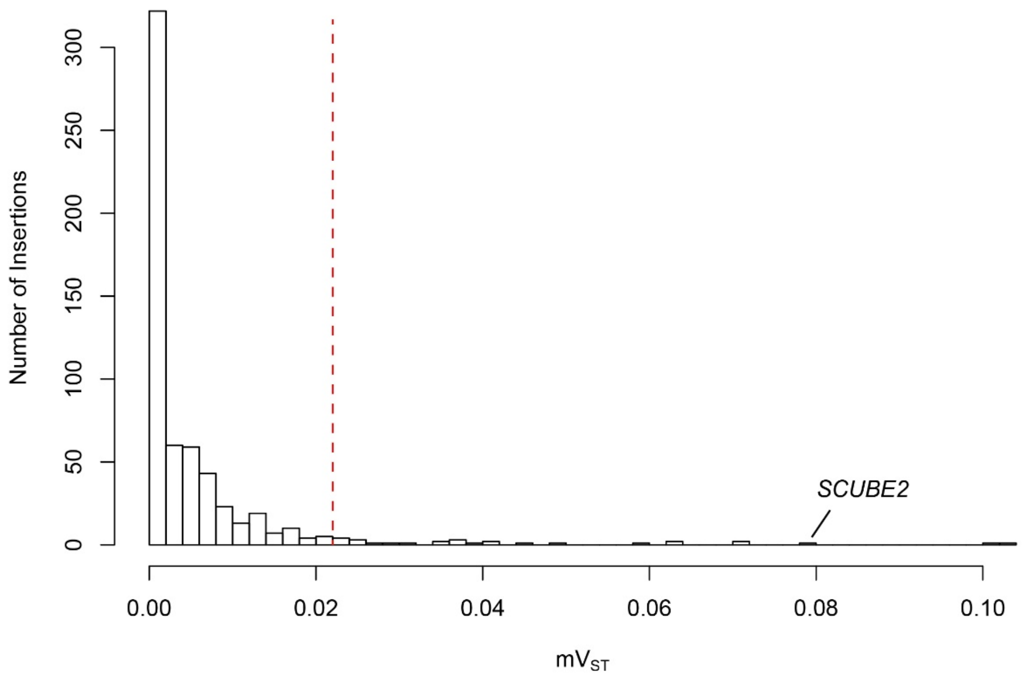

Supplementary Fig. 12 | mV_{ST} distribution of non-repetitive insertions between Tibetan and Han Chinese. The red-dashed line refers to the top 5% of 593 candidate non-repetitive insertions.

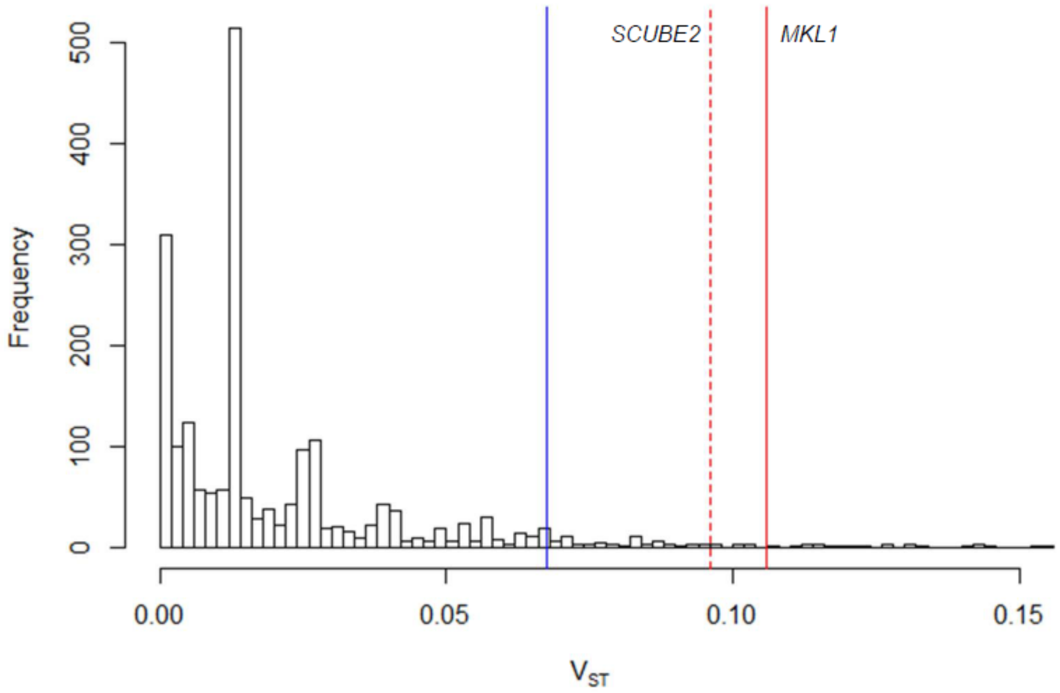

Supplementary Fig. 13 | Null distribution of V_{ST} between the Tibetan and Han Chinese populations.

The histogram represents the V_{ST} between the Tibetan and Han Chinese under the simulation model without natural selection (Suplementary Figure 18). The solid blue line shows the 95 percentiles of this null distribution (V_{ST}=0.0675). The solid and the dash red line corresponds to the observed V_{ST} of the *MKL1* deletion (V_{ST}=0.106, corresponding to 98.7 percentile of the null distribution) and the *SCUBE2* insertion (V_{ST}[CN]=0.096, corresponding to 98.3 percentile in the null distribution; note that mVST=min(V_{ST}[CN], V_{ST}[norm-RD]) and the V_{ST}[norm-RD]=0.079, therefore mV_{ST}=0.079 for this insertion) in the real data respectively.

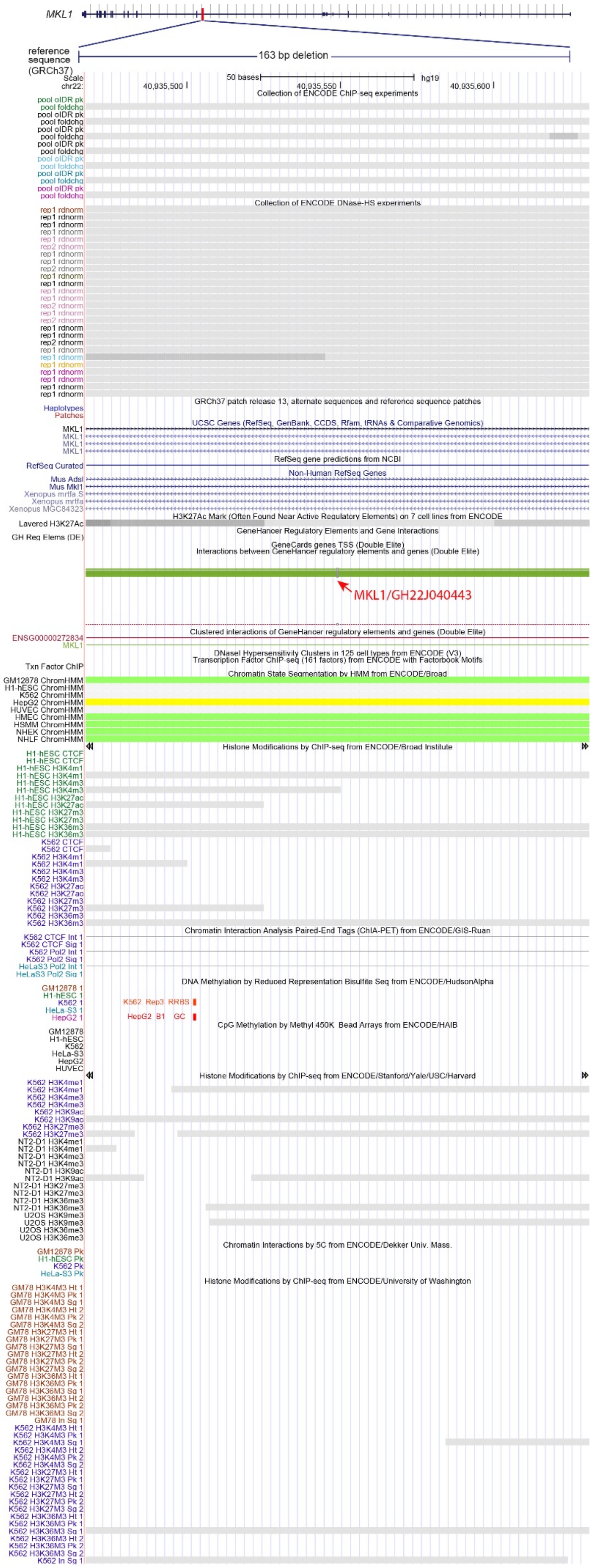

Supplementary Fig. 14 | The epigenetic signals overlapped with the *MKL1* 163-bp deletion. The data was obtained from ENCODE at UCSC Genome Browser.

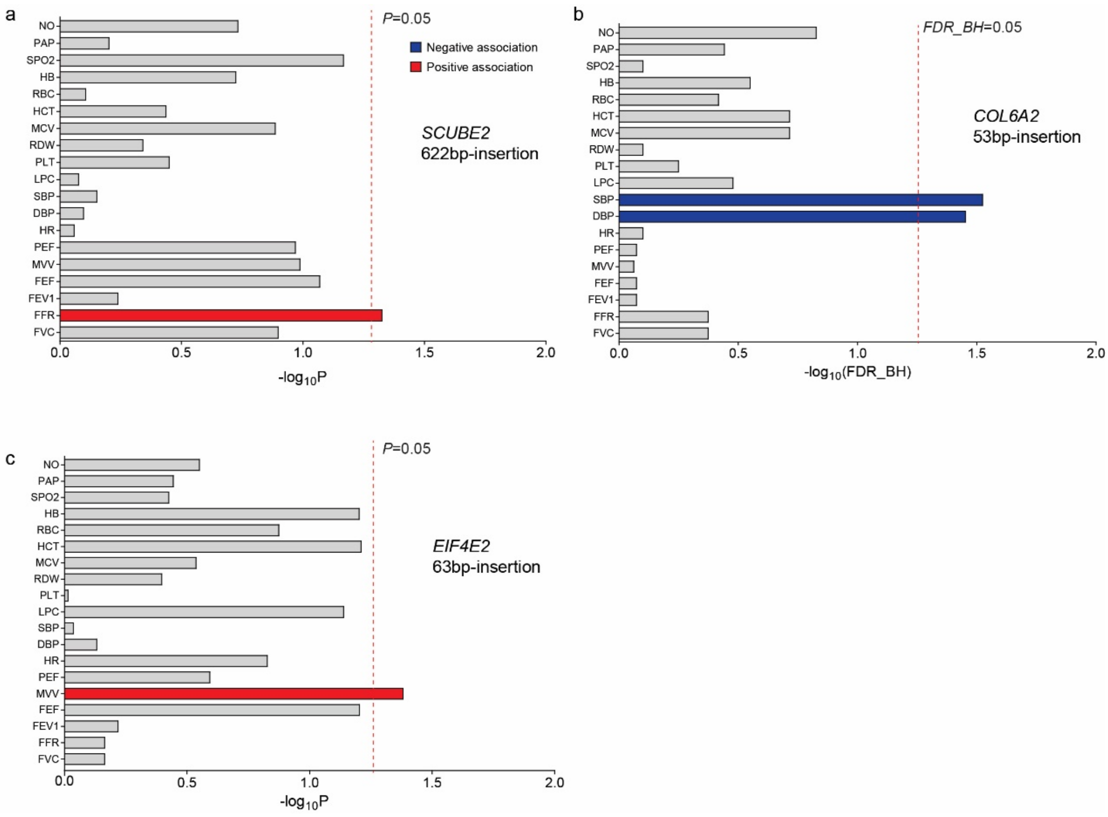

Supplementary Fig. 15 | Genetic association analysis between three candidate SVs and multiple physiological traits in Tibetans. Dot line in red refers to significance cutoff (FDR=0.05 for b; P=0.05 for a. and c.), see Methods for the abbreviation of each phenotype. Blue- and red-filled histogram refers to negative and positive associated relevance, respectively.

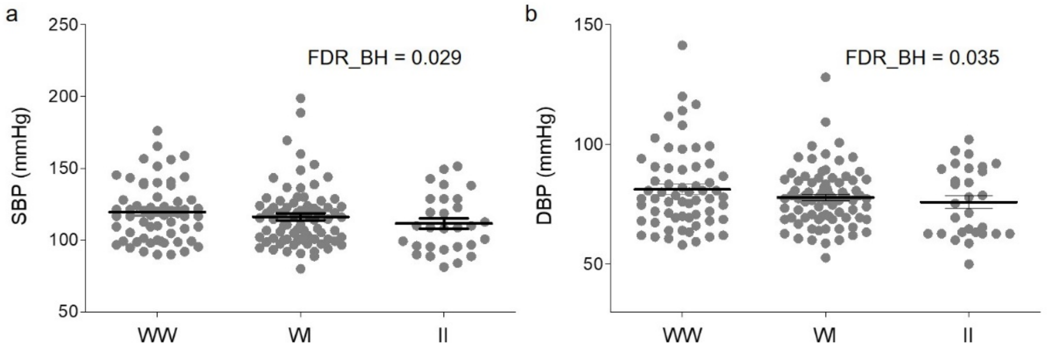

Supplementary Fig. 16 | Comparison of SBP and DBP among three genotypes of the *COL6A2* insertion. I: 53bp insertion in *COL6A2*; W: wildtype.

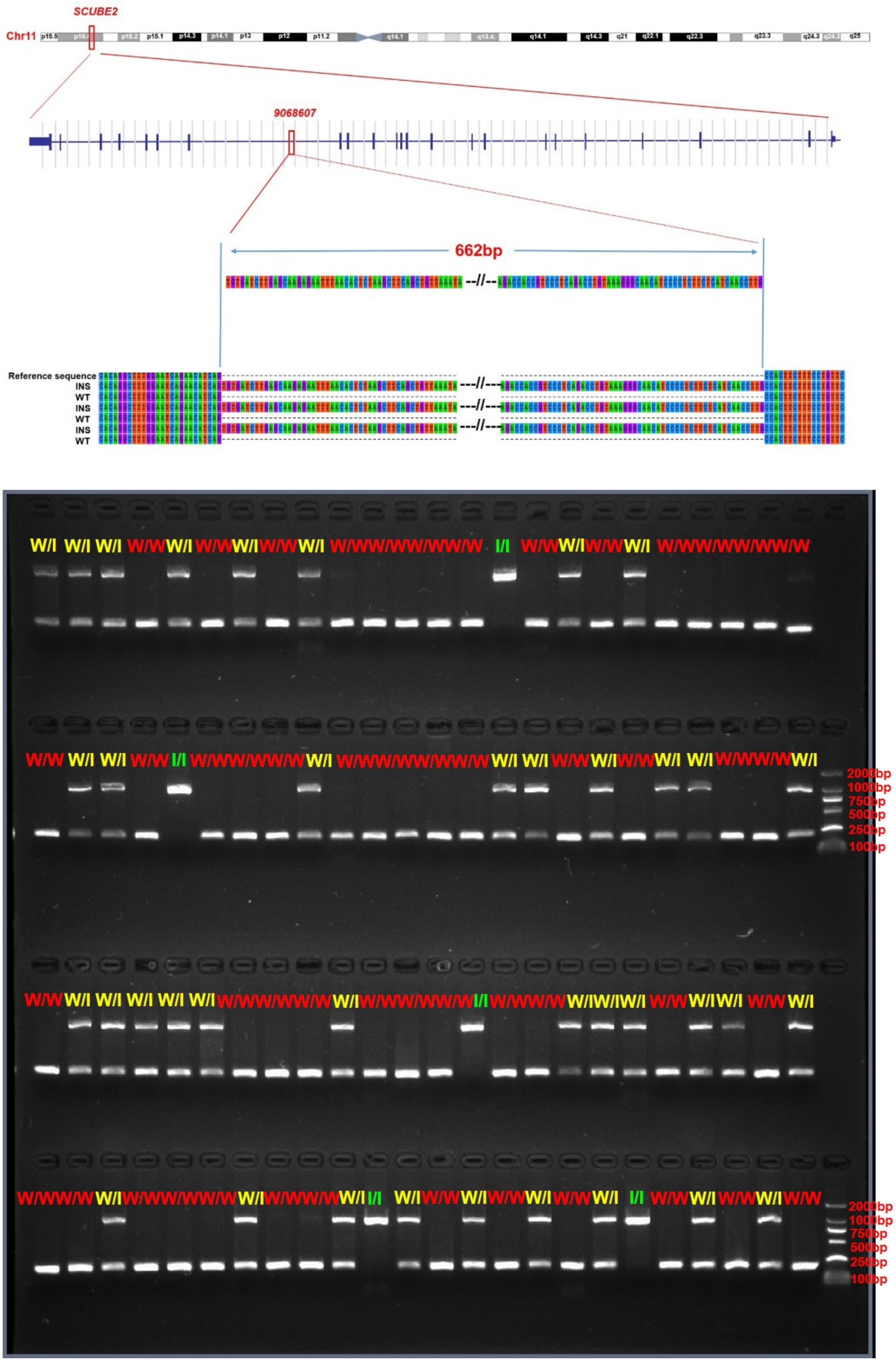

Supplementary Fig. 17 | Overview of insertion at *SCUBE2* and validation. Sanger PCR validation of insertion region were aligned as follow (up); agarose gel electrophoresis evaluation for 662bp-insertion at SCUBE2 (down). “W”: wildtype; “I”: insertion.

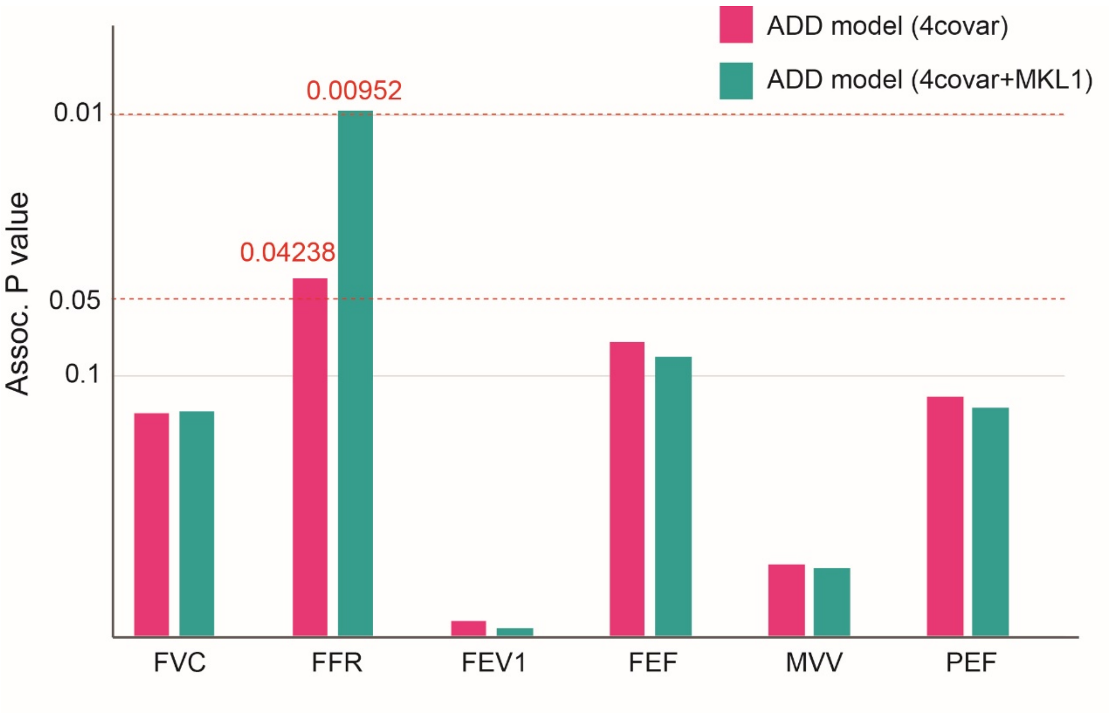

Supplementary Fig. 18 | Comparison of the P values of associations with lung functions using two different models. The red bars indicate the P values using only the *SCUBE2* 622bp-insertion, and the green bars indicate the P values when including both the *SCUBE2* 622bp-insertion and the *MKL1* 163bp-delection.

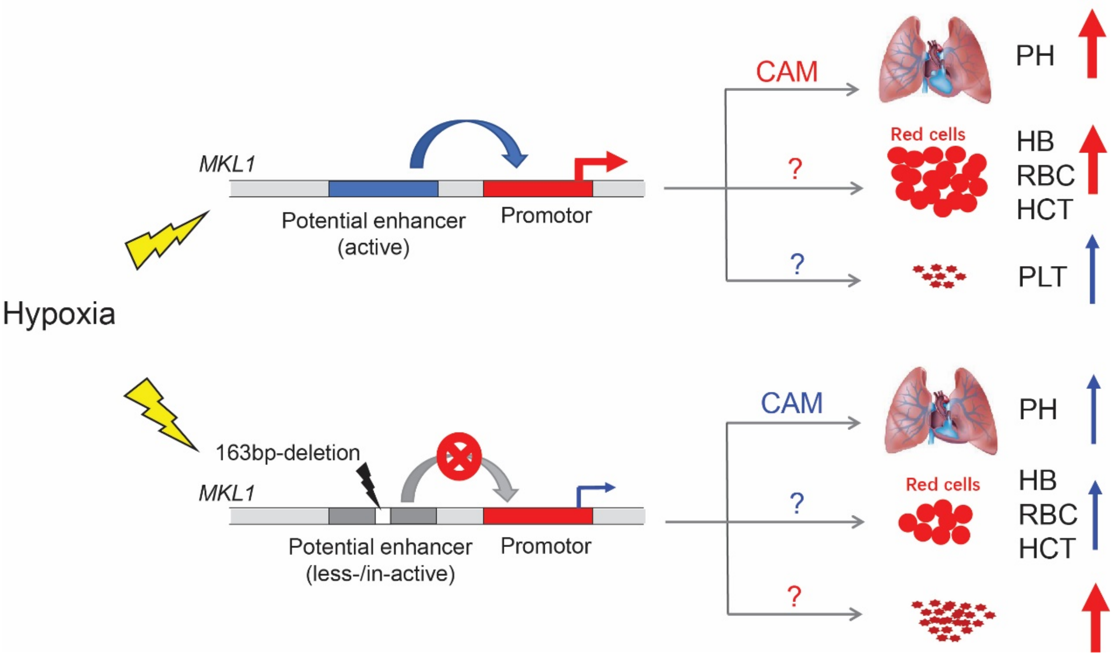

Supplementary Fig. 19 | Schematic diagram of the putative effects of the *MKL1* 163bp-deletion on the associated phenotypes. CAM: cell adhesion molecules; PH: pulmonary hypertension; HB: hemoglobin; RBC: red blood cell; HCT: hematocrit; PLT: platelet. The regulatory pathway from CAM to PH were based on the previous studies [41, 42].

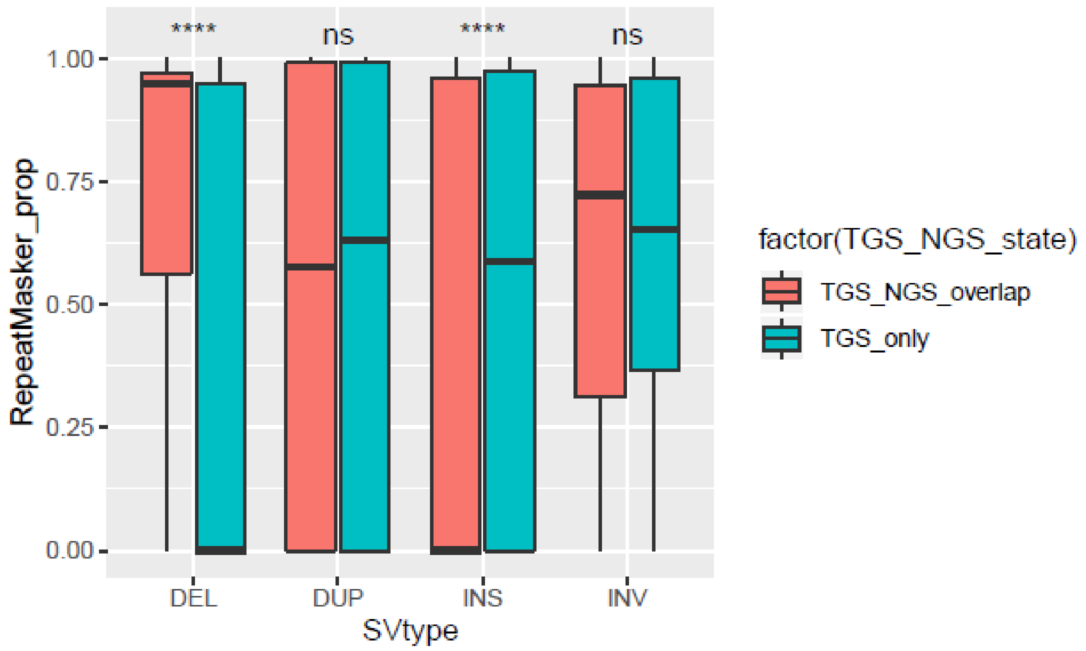

Supplementary Fig. 20. Comparison of repeat element proportion between TGS-only SVs and NGS recalled SVs. The proportion of repeat element is compared between the SVs only detected by TGS (TGS-only) and the SVs detected by both TGS and NGS (TGS_NGS_overlap) for each of the SV type. The repeat element was annotated by RepeatMasker. ****: $P<0.0001$; ns: non-significant.

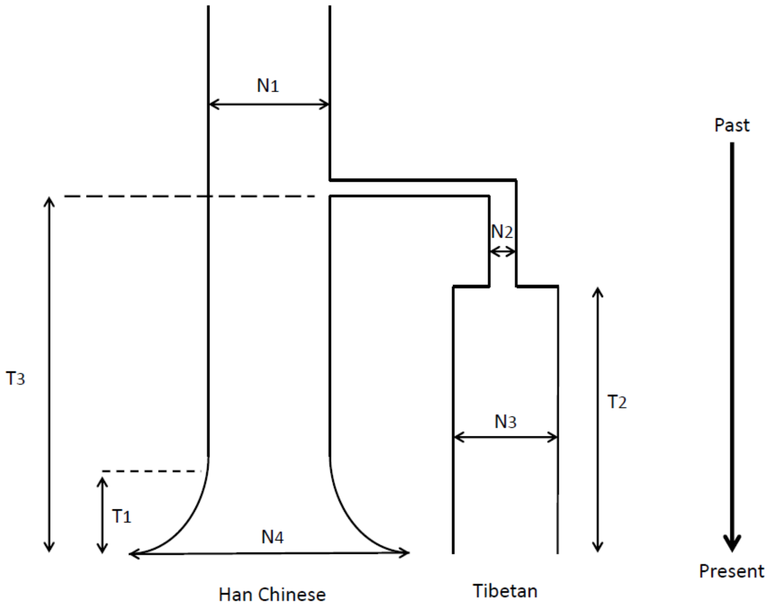

Supplementary Fig. 21 | Model of simulation for the null V_{ST} distribution between the Tibetan and Han Chinese without natural selection.

Following our previous estimation [26], we assumed that the Tibetan and the Han Chinese split 10,000 years ago (*T3*), and after the divergence a bottleneck event in the Tibetan occurred till 9,000 years before present (*T2*). We also assumed an exponential growth of effective population size (*Ne*) for Han Chinese starting at 2,000 years before present (*T1*). We set the *Ne* of the Tibetan-Han Chinese common ancestry (*N1*) to be 20,000, the *Ne* at *T2* in Tibetan to be 5,000, and the *Ne* for present Tibetan and Han Chinese at *T3* to be 20,000 (*N3*) and 50,000 (*N4*) respectively.

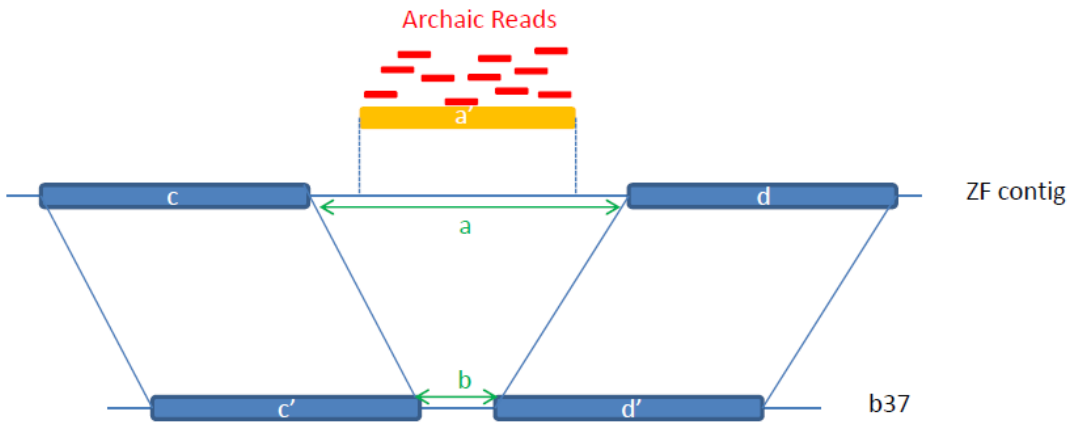

Supplementary Fig. 22 | Illustration of novel sequence position identification on the reference genome.

The blue boxes represent the alignments of ZF1 and GRCh37 (c and c'; d and d'). The gaps between adjacent alignments are indicated in green (a, b). The red bars represent the archaic reference unmapped reads, and the orange rectangle indicates the region of these unmapped reads (a').
